# Author Correction: Thorium–phosphorus triamidoamine complexes containing Th–P single- and multiple-bond interactions

**DOI:** 10.1038/s41467-023-38917-4

**Published:** 2023-05-26

**Authors:** Elizabeth P. Wildman, Gábor Balázs, Ashley J. Wooles, Manfred Scheer, Stephen T. Liddle

**Affiliations:** 1 School of Chemistry, The University of Manchester, Oxford Road, Manchester, M13 9PL UK; 2 Institute of Inorganic Chemistry, University of Regensburg, Universitaets str.31, Regensburg, 93053 Germany

Correction to: *Nature Communications* 10.1038/ncomms12884, published online 29 September 2016

The original version of this Article contained errors in the ^31^P NMR chemical shifts of compounds **3** and **5**, due to an instrument referencing error that has been recognized later. For compound **3**, an incorrect ^31^P NMR chemical shifts value of 150.8 ppm was reported in the 3^rd^ sentence of the 3^rd^ paragraph of the Synthesis subsection of the Results, in the first entry of the second line of Table 1, and in the 6^th^ sentence of the 4^th^ paragraph of the Discussion; an incorrect value of 150.79 ppm was reported in the 12^th^ sentence of the section ‘Preparation of [Th(Tren^TIPS^)(PH)][Na(12C4)_2_] (**3**)’ of the Methods . The correct version replaces 150.8 and 150.79 with 198.8. For compound **5**, an incorrect ^31^P NMR chemical shifts value of 24.5 ppm was reported in the 4^th^ sentence of the 4^th^ paragraph of the Synthesis subsection of the Results, in the first entry of the third line of Table 1, and in the 6^th^ sentence of the 4^th^ paragraph of the Discussion; an incorrect value of 24.46 ppm was reported in the 14^th^ sentence of the section ‘Preparation of [{Th(Tren^TIPS^)}2(μ-PH)] (**5**).’ of the Methods. The correct version replaces 24.5 and 24.46 with 145.7. This has been corrected in both the PDF and HTML versions of the Article.

