# Supplementary material for: Thorium–phosphorus triamidoamine complexes containing Th–P single- and multiple-bond interactions
Source: Nat Commun. 2016 Sep 29;7:12884. doi: 10.1038/ncomms12884 (PMC5056418; doi:10.1038/ncomms12884)
Supplement: Supplementary Information — Supplementary Figures 1-25, Supplementary Tables 1-4, Supplementary Methods and Supplementary References [file ncomms12884-s1.pdf]

Supplementary Figures

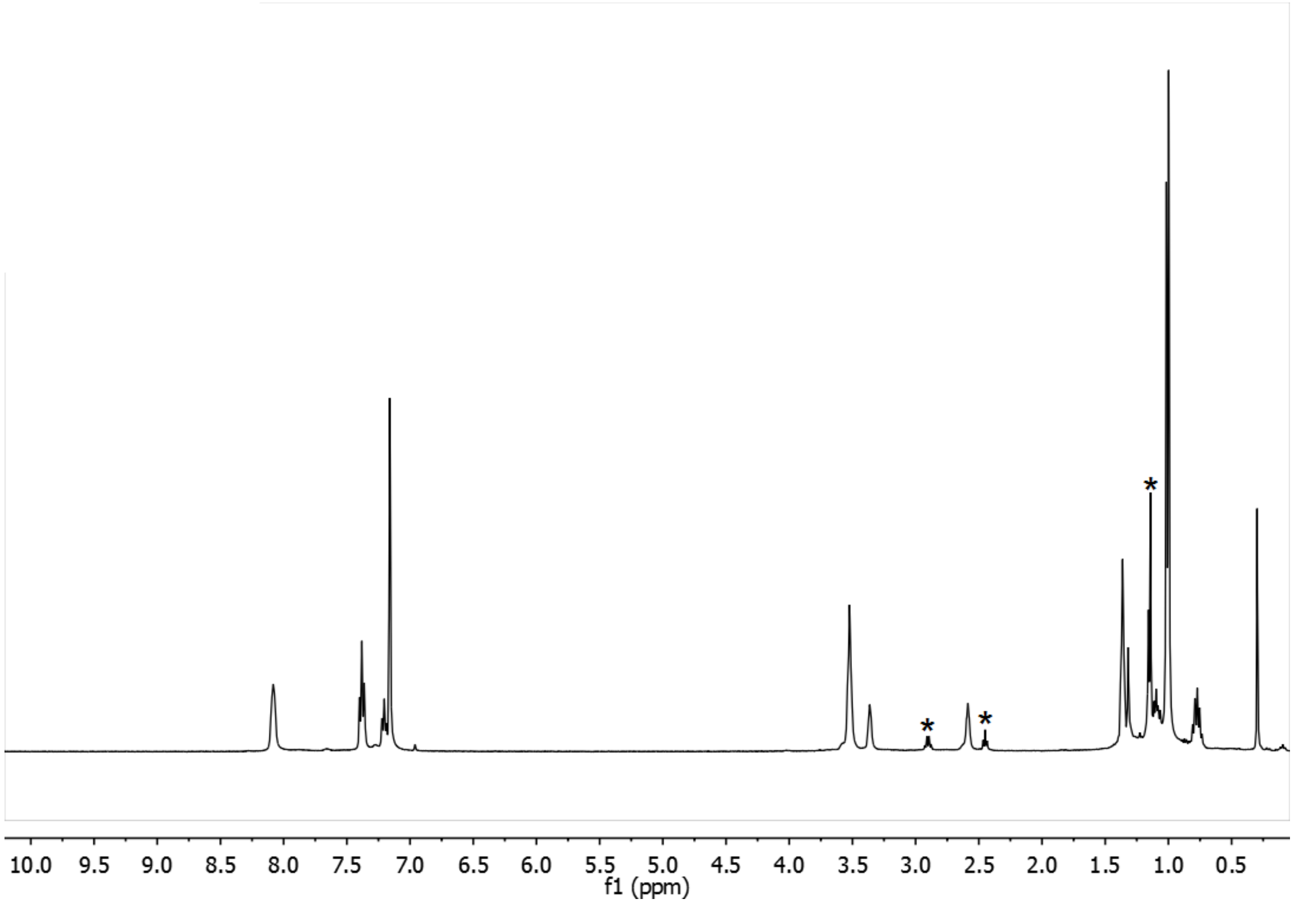

***Supplementary Figure 1.** ^1H NMR spectrum of isolated $[Th(Tren^{TIPS})(OCH_2CH_2CH_2CH_2NEt_3)][BPh_4]$. * = $Tren^{TIPS}H_3$ impurity.*

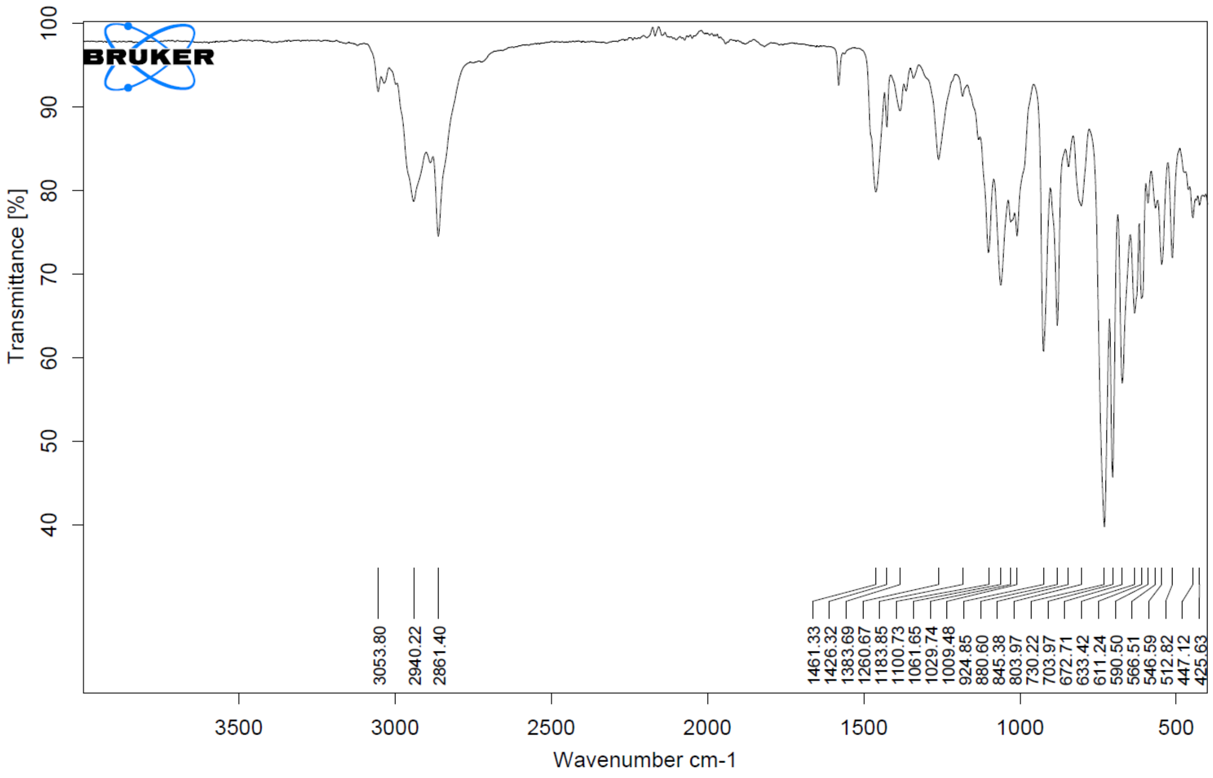

Supplementary Figure 2. ATR-IR spectrum of isolated [Th(Tren^{TIPS})(OCH$_2$CH$_2$CH$_2$CH$_2$NEt$_3$)][BPh$_4$].

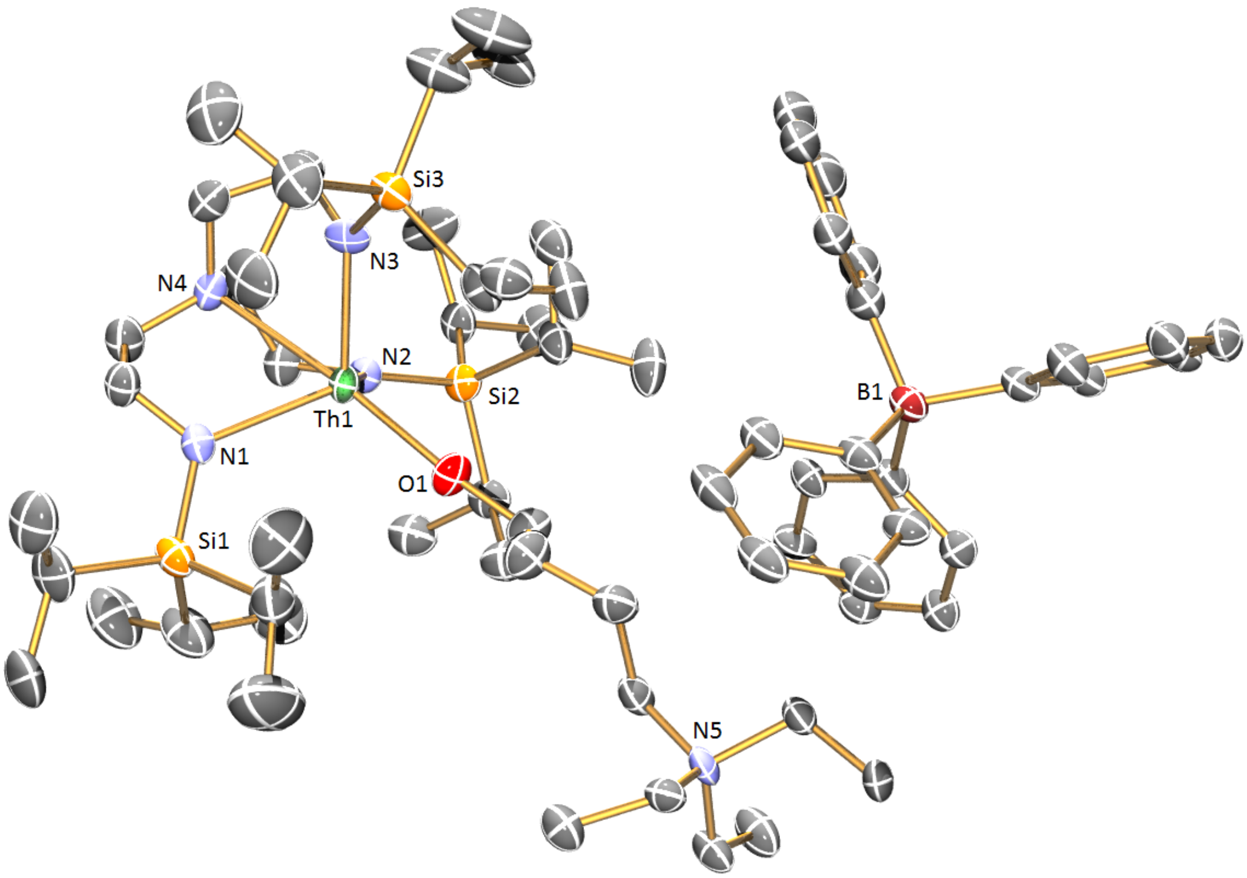

Supplementary Figure 3. Molecular structure of [Th(Tren^{TIPS})($OCH_2CH_2CH_2CH_2NEt_3$)][BPh_4] at 150 K. Displacement ellipsoids set to 40% and hydrogen atoms are omitted for clarity.

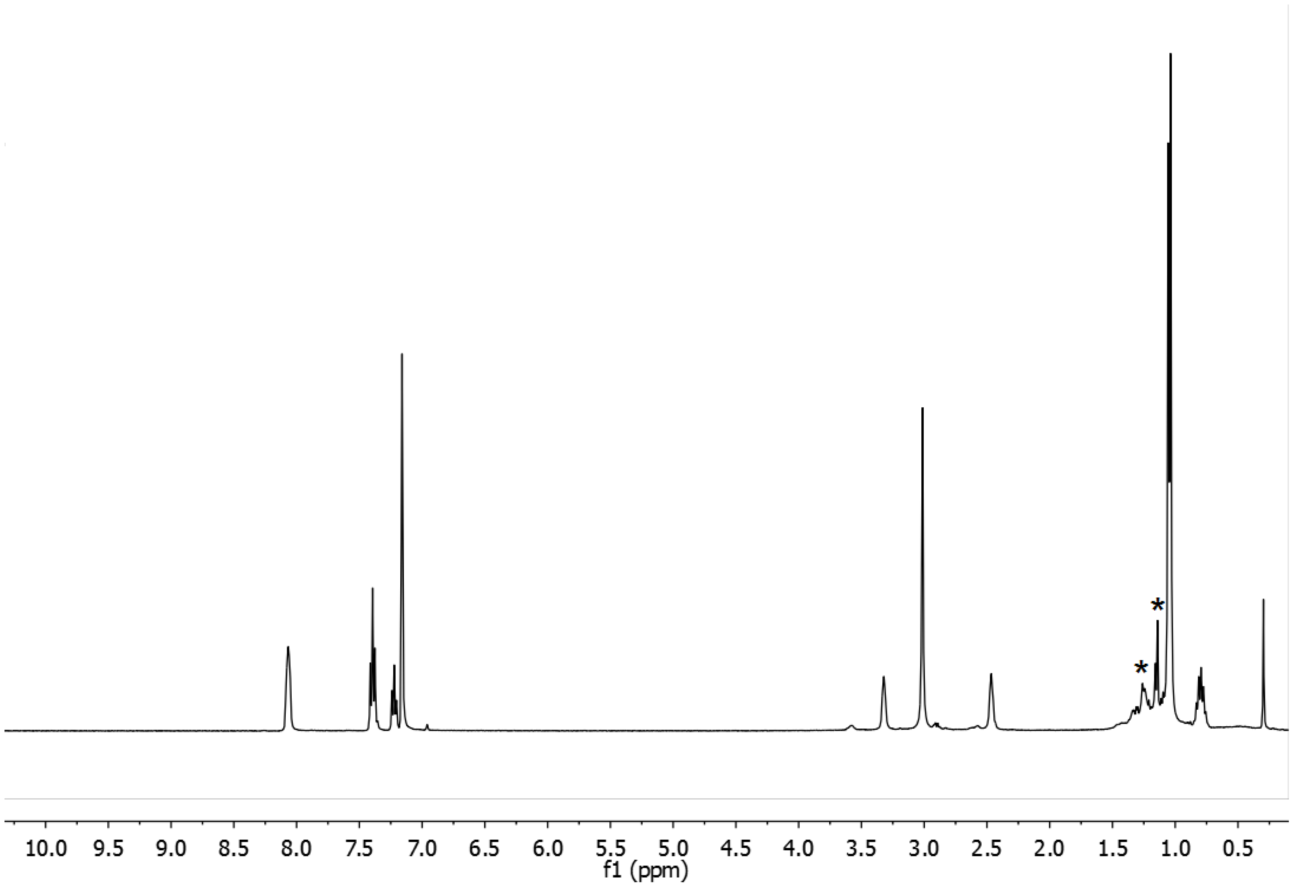

***Supplementary Figure 4.** ^{1}H NMR spectrum of isolated [Th(Tren^{TIPS})(DME)][BPh_4] (1). * = Tren$^{TIPS}H_3$ impurity.*

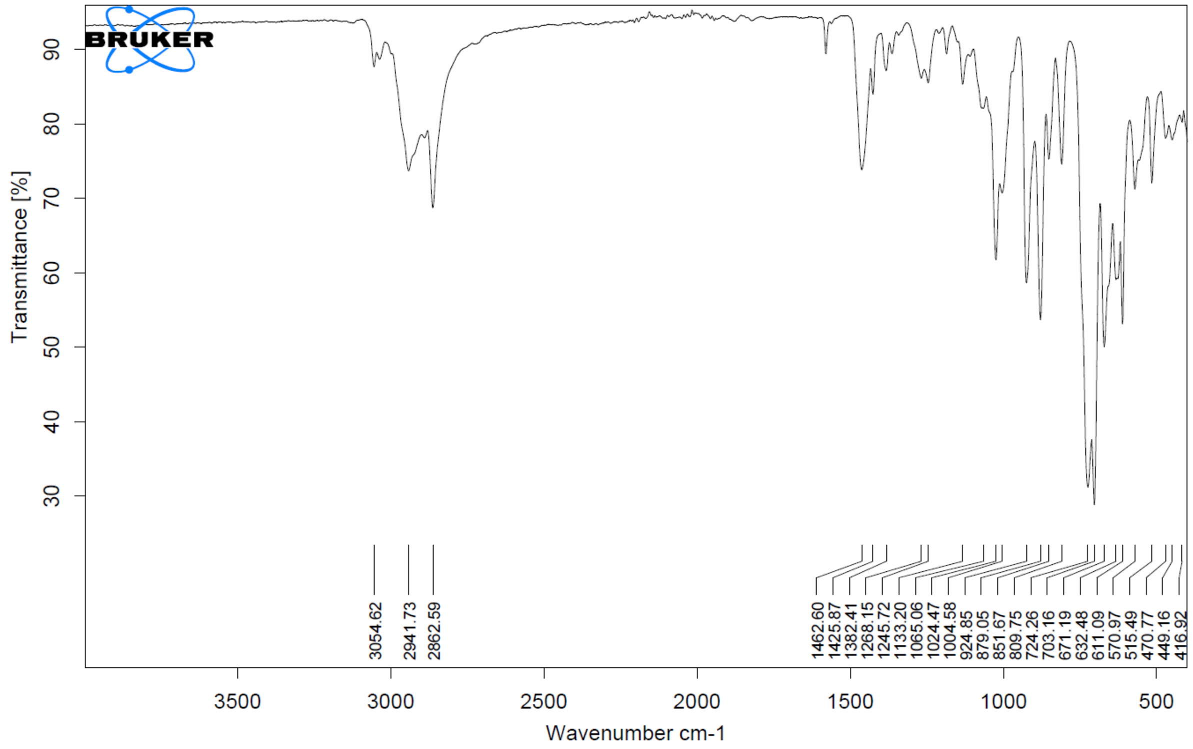

Supplementary Figure 5. ATR-IR spectrum of isolated [Th(Tren^{TIPS})(DME)][BPh$_4$] (1).

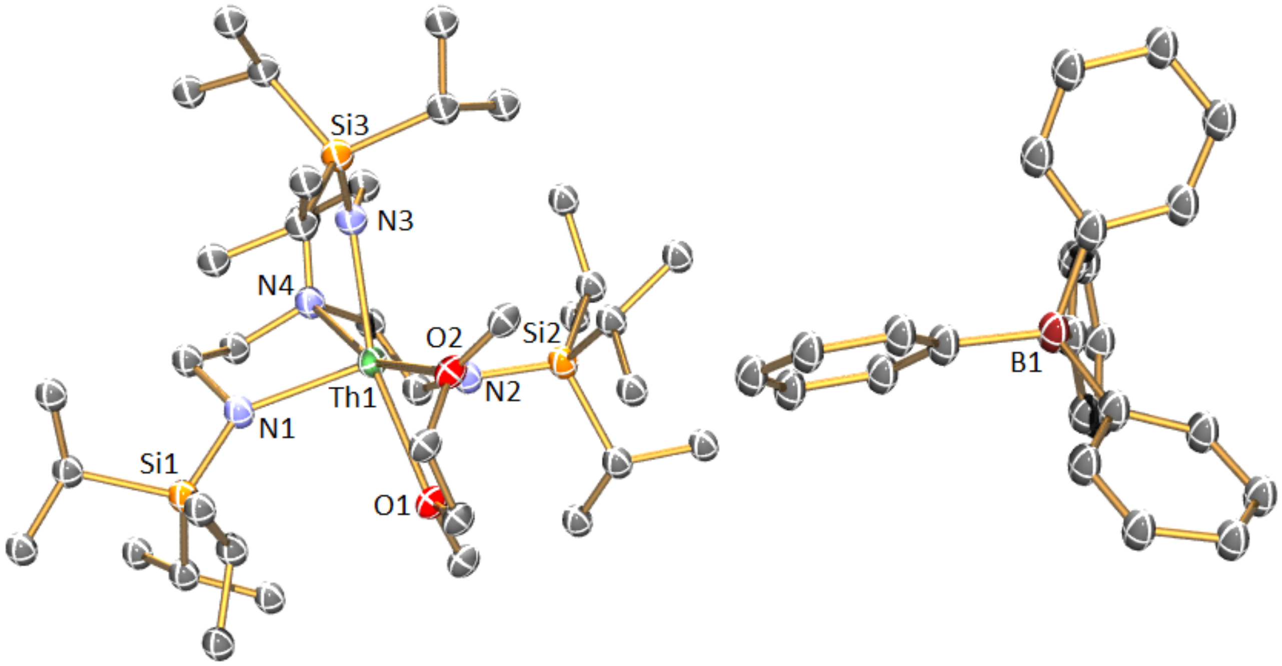

Supplementary Figure 6. Molecular structure of [Th(Tren^{TIPS})(DME)][BPh$_4$] (1) at 150 K. Displacement ellipsoids set to 40% and hydrogen atoms are omitted for clarity.

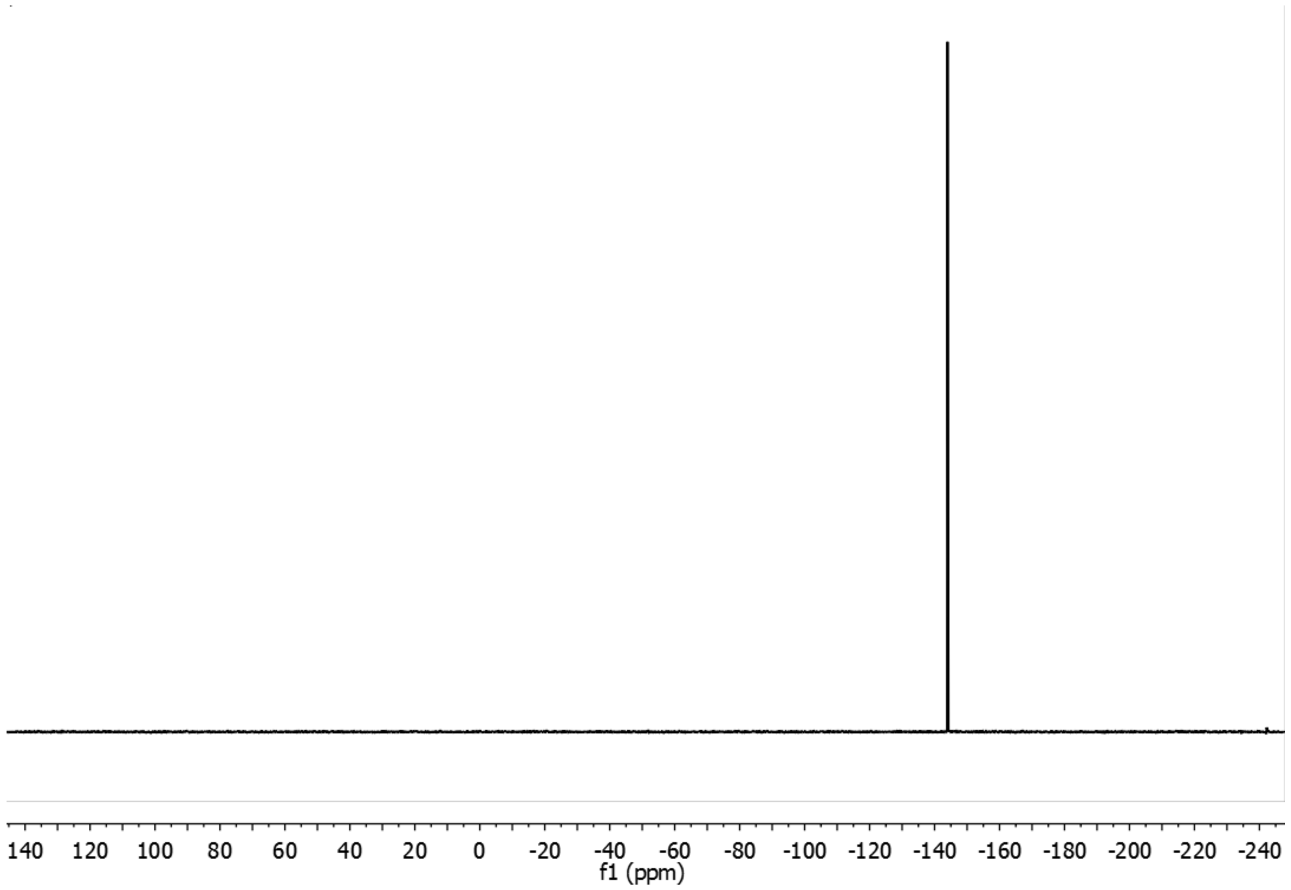

Supplementary Figure 7.** $^{31}P\{^1H\}$ **NMR spectrum of isolated [Th(Tren^{TIPS})(PH_2)] (2).

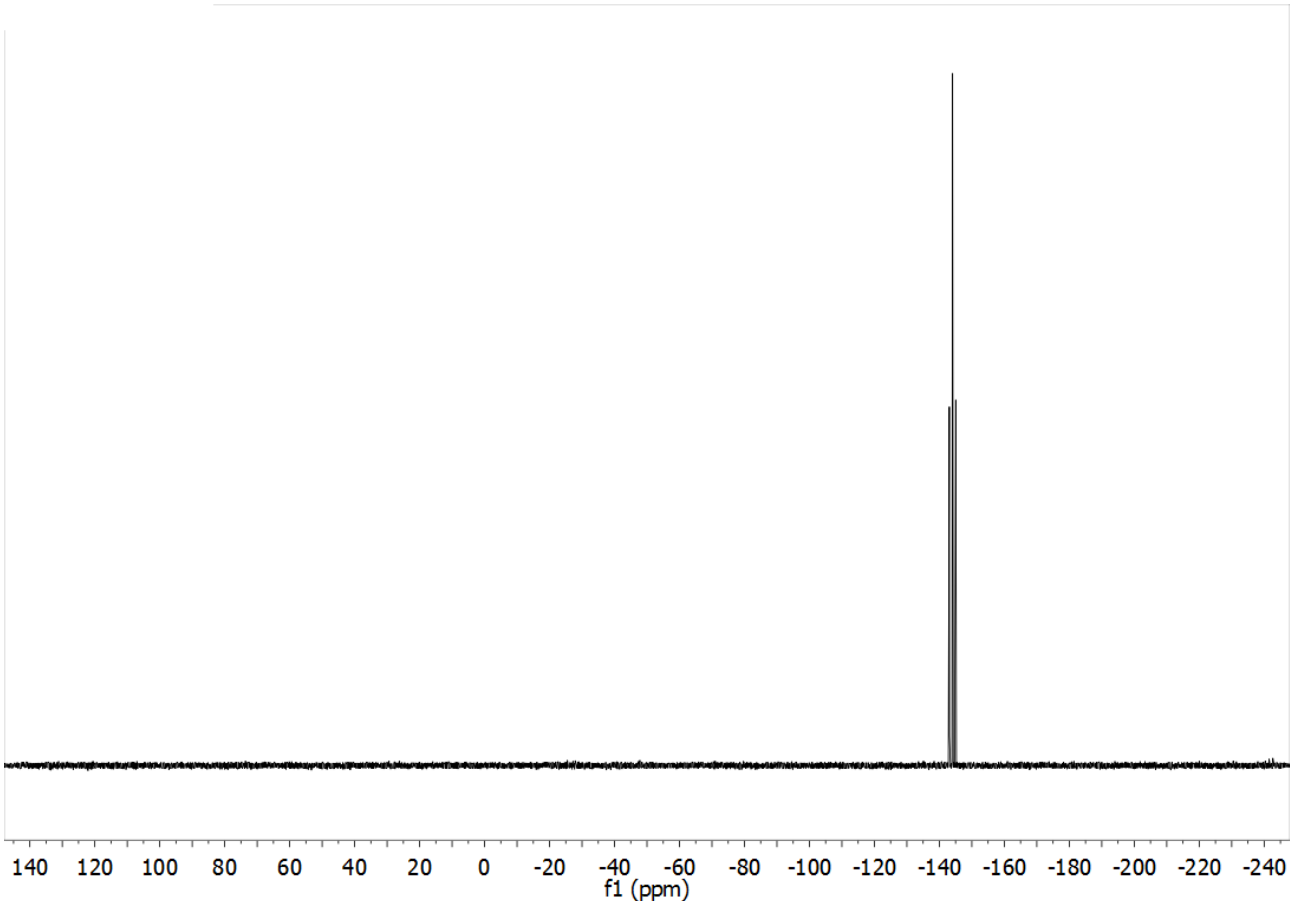

Supplementary Figure 8. ***^{31}P NMR spectrum of isolated [Th(Tren^{TIPS})(PH_2)] (2).***

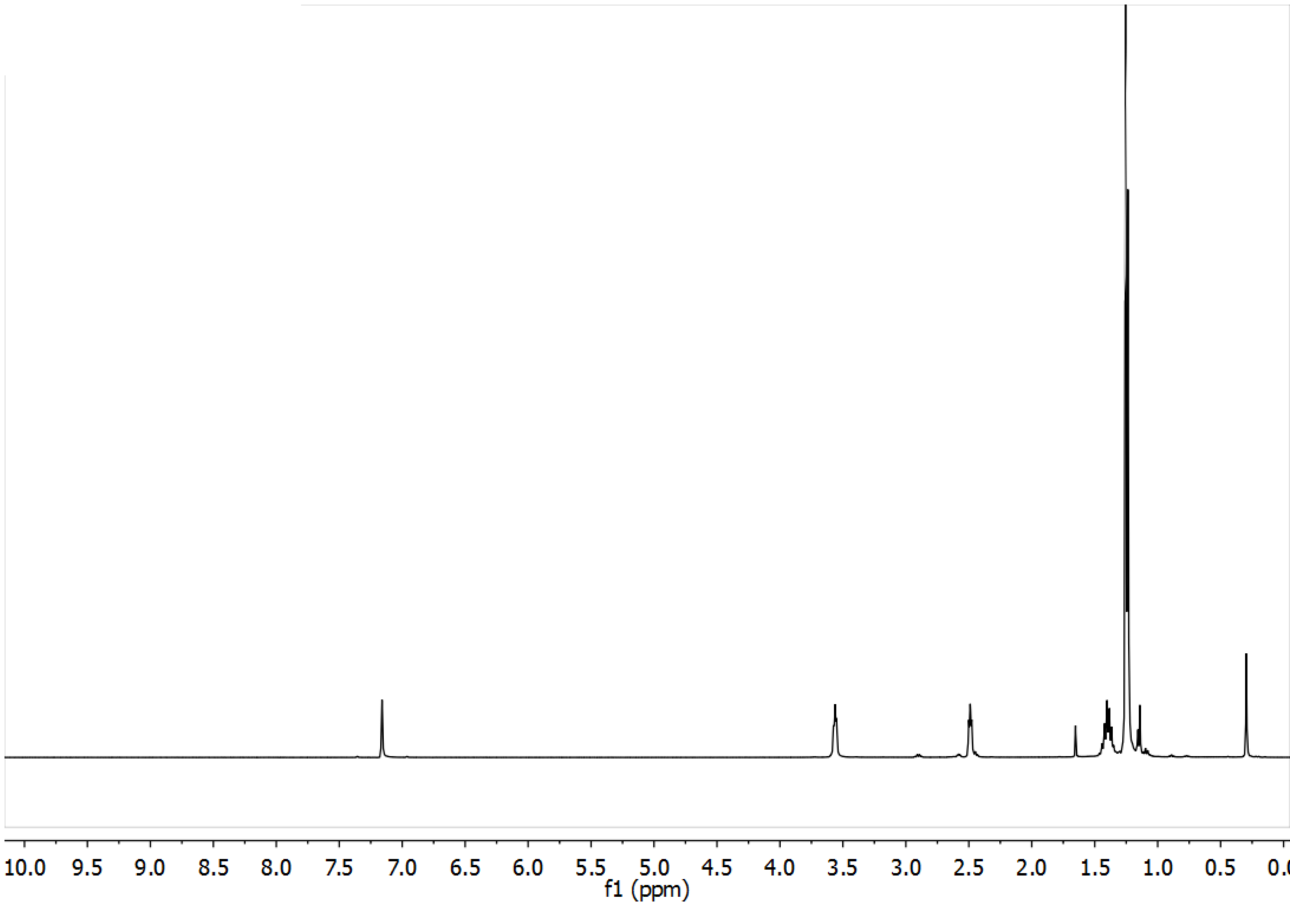

Supplementary Figure 9. ***^{1}H NMR spectrum of isolated [Th(Tren^{TIPS})(PH_2)] (2).***

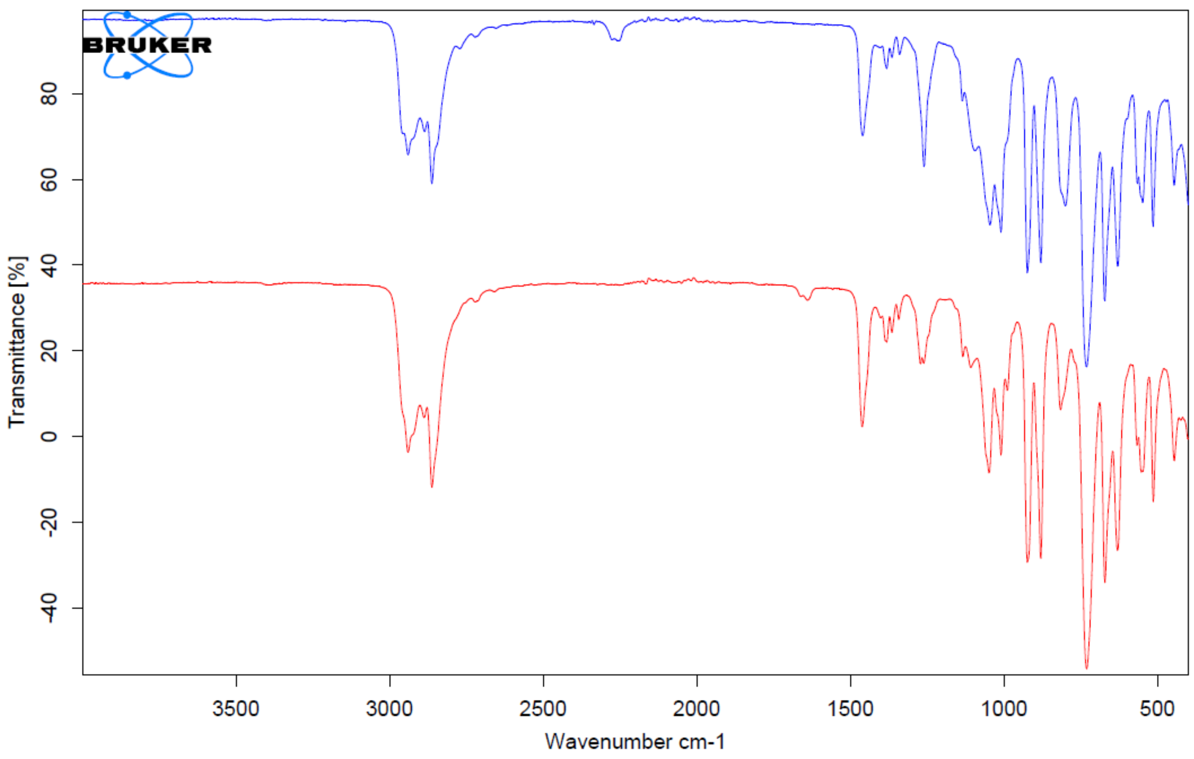

Supplementary Figure 10. ATR-IR spectra of isolated [Th(Tren^{TIPS})(PH_2)] (2, blue) and [Th(Tren^{TIPS})(PD_2)] (2D, red). The transmittance scale is indicative and relative, not absolute.

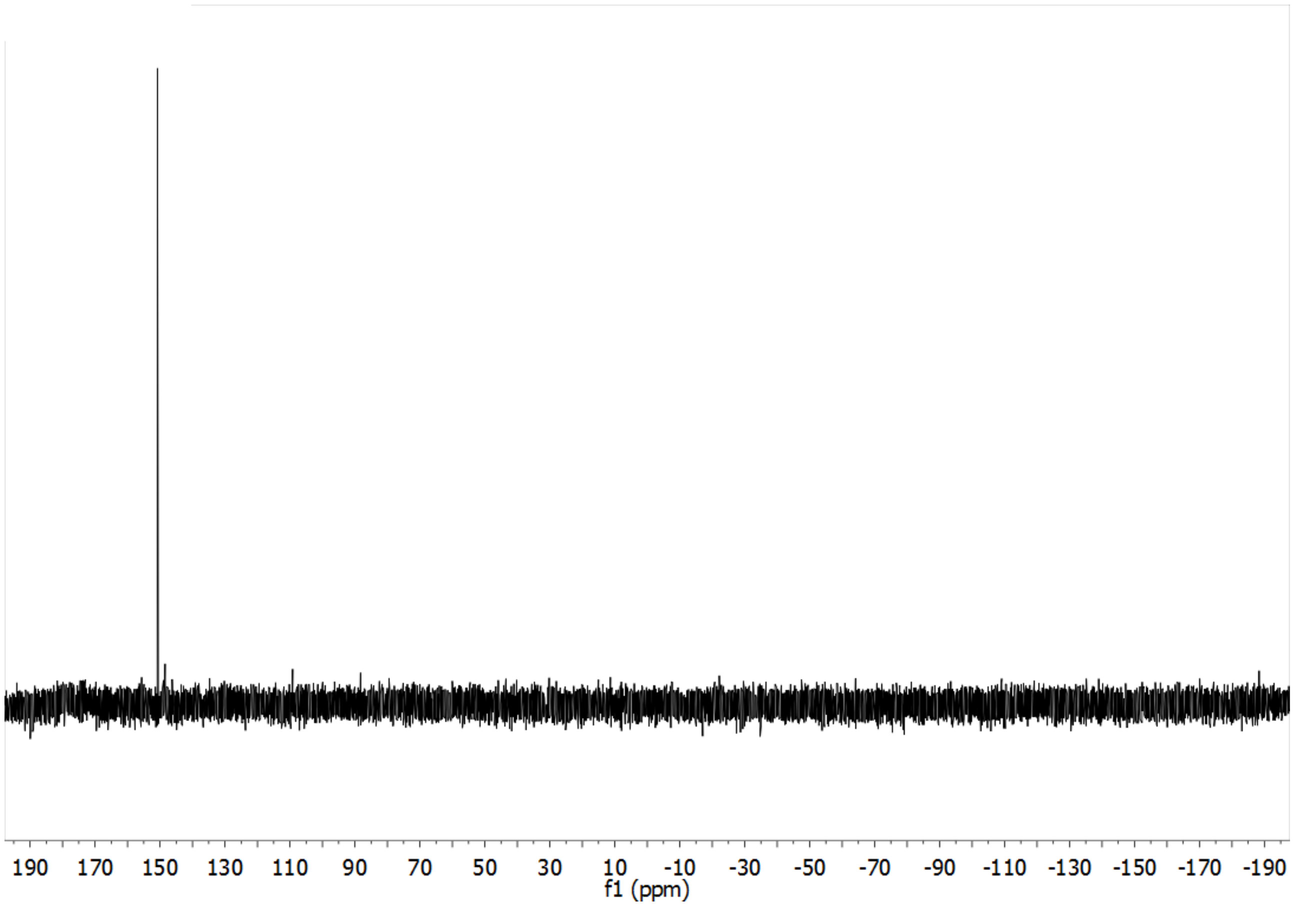

Supplementary Figure 11. ***$^{31}P\{^{1}H\}$ NMR spectrum of isolated $[Th(Tren^{TIPS})(PH)][Na(12C4)_2]$ (3).***

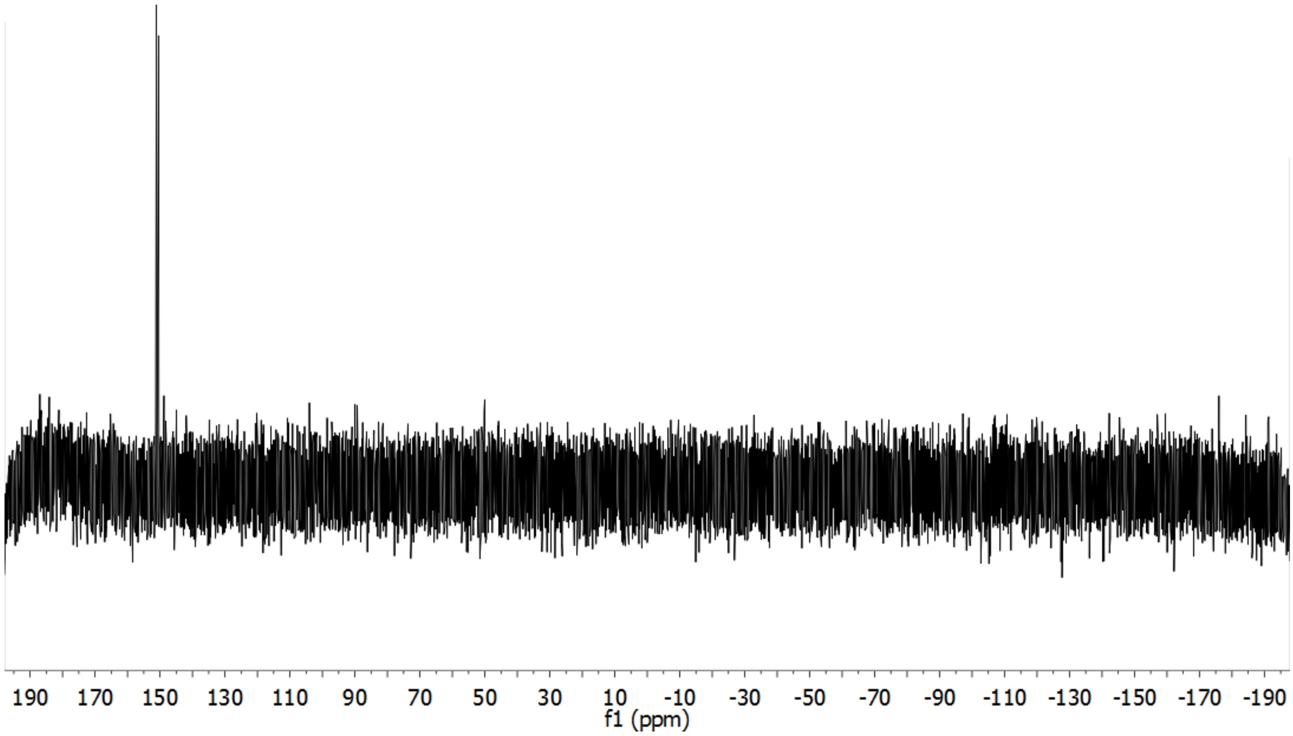

Supplementary Figure 12. ***^{31}P NMR spectrum of isolated [Th(Tren^{TIPS})(PH)][Na(12C4)$_2$] (3).***

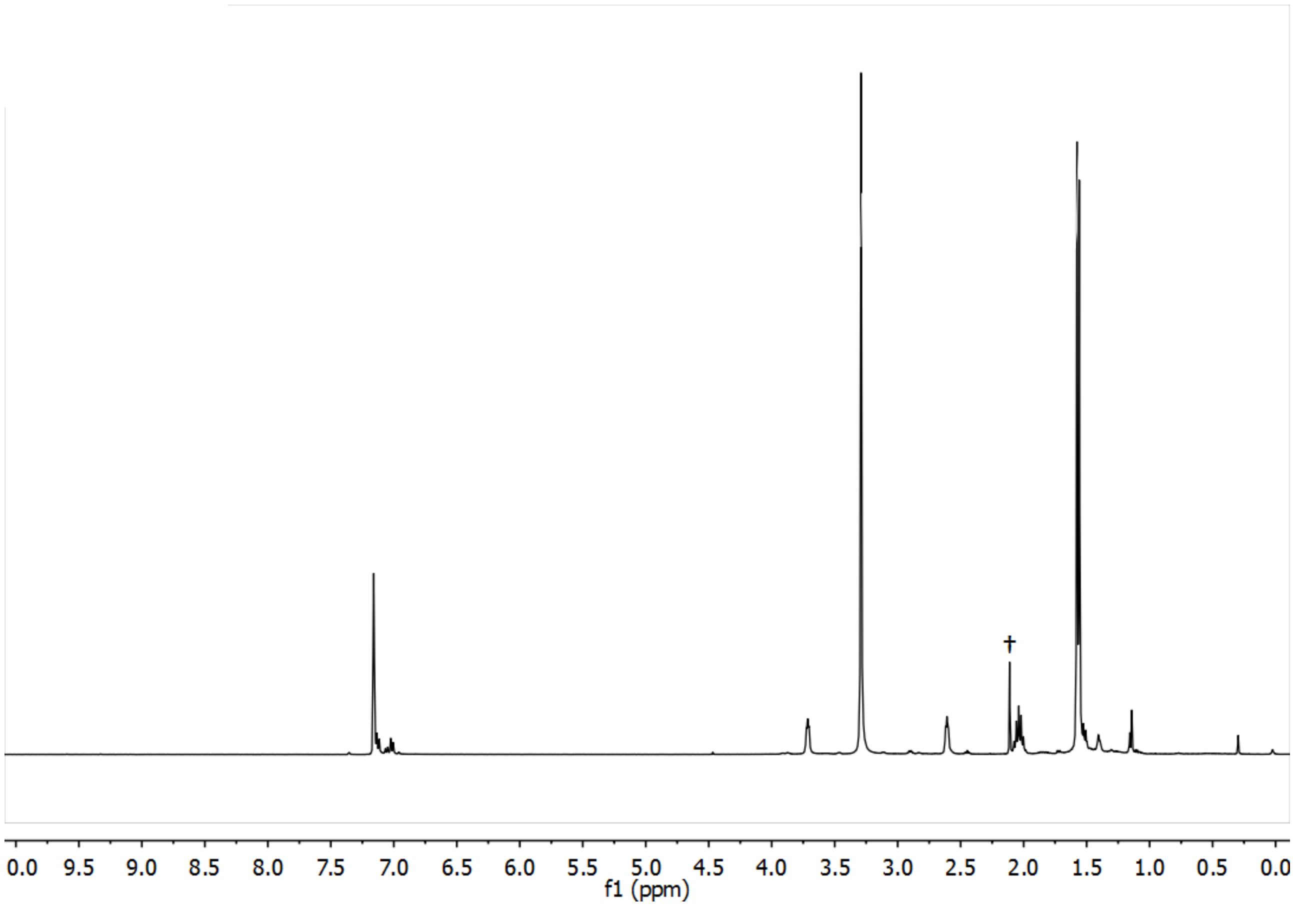

Supplementary Figure 13. ***^{1}H NMR spectrum of isolated [Th(Tren^{TIPS})(PH)][Na(12C4)$_2$] (3). † = residual solvent resonance.***

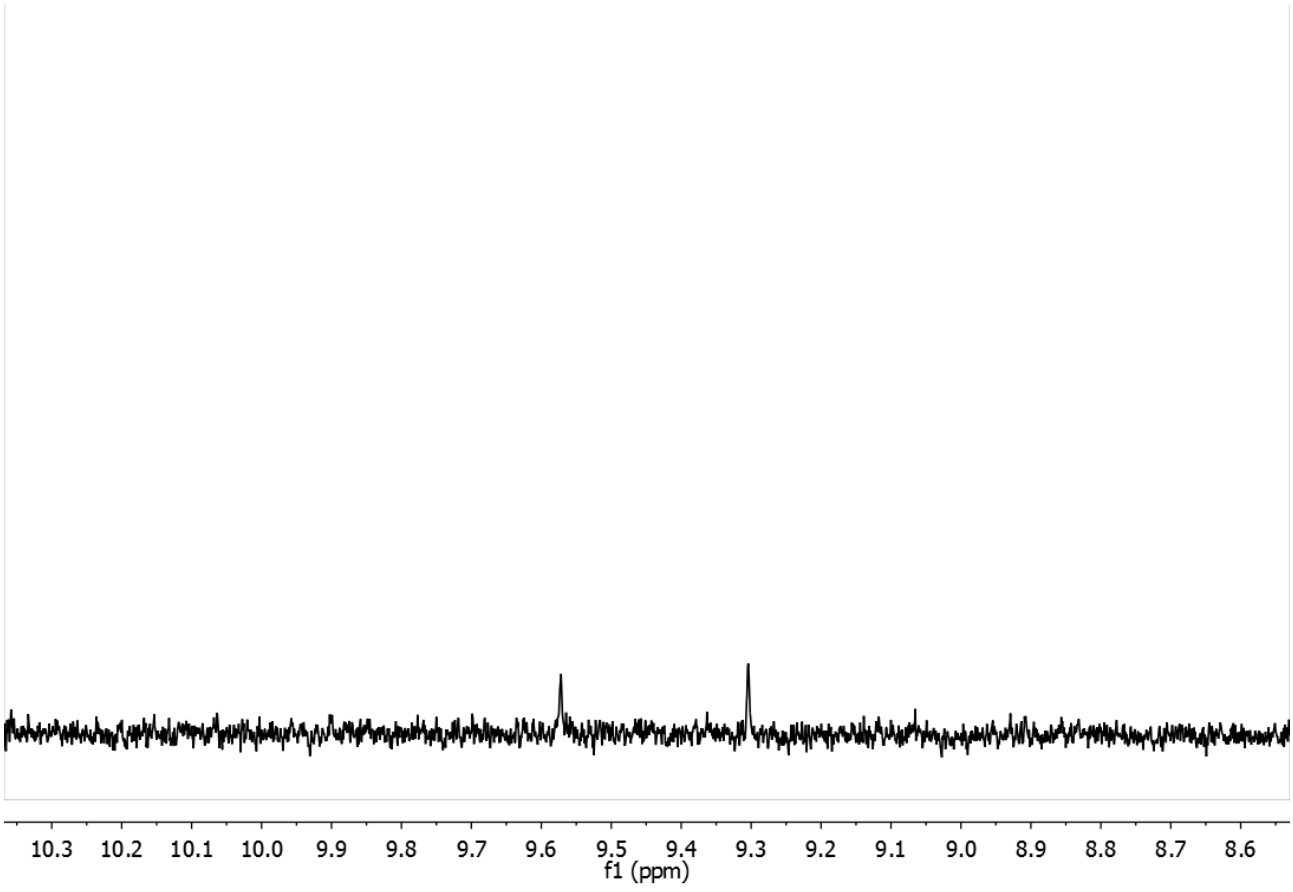

Supplementary Figure 14. Zoom in of 8.6-10.3 region of the ^{1}H NMR spectrum of isolated [Th(Tren^{TIPS})(PH)][Na(12C4)$_2$] (3) showing the P-H resonance.

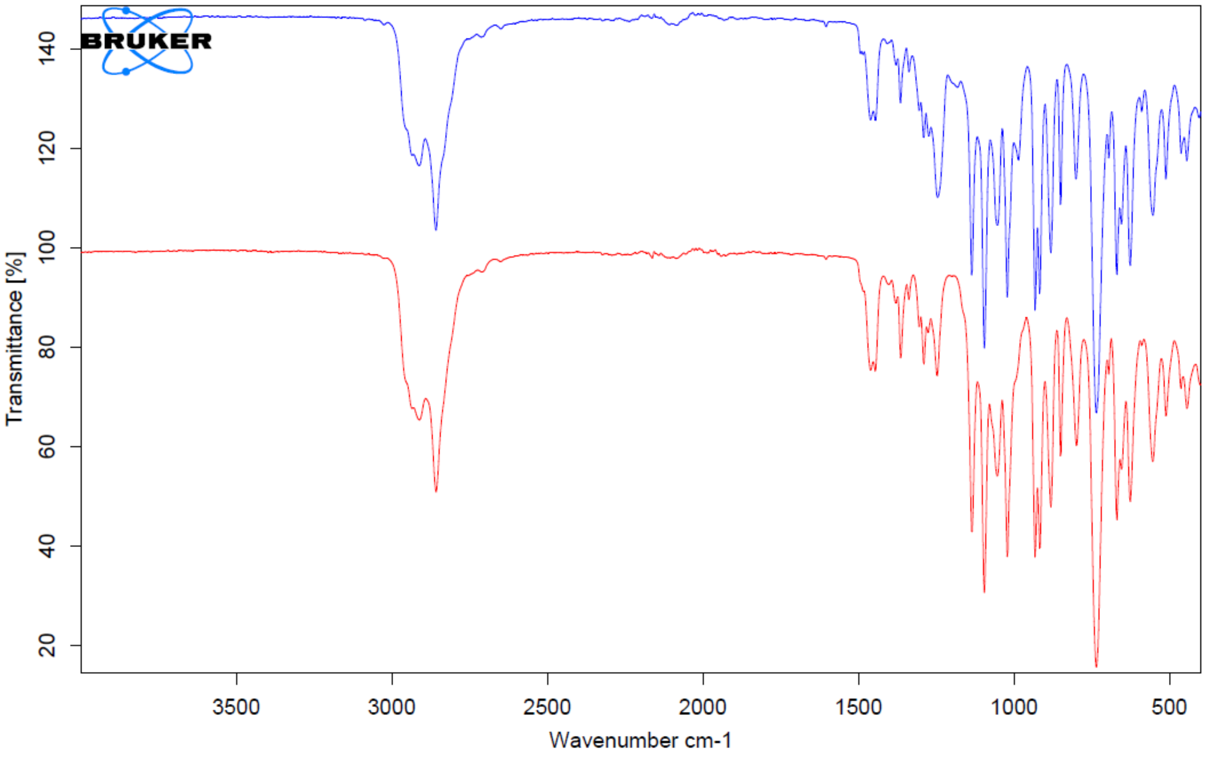

Supplementary Figure 15. ATR-IR spectrum of isolated [Th(Tren^{TIPS})(PH)][Na(12C4)$_2$] (3, blue) and [Th(Tren^{TIPS})(PD)][Na(12C4)$_2$] (3D, red). The transmittance scale is indicative and relative, not absolute.

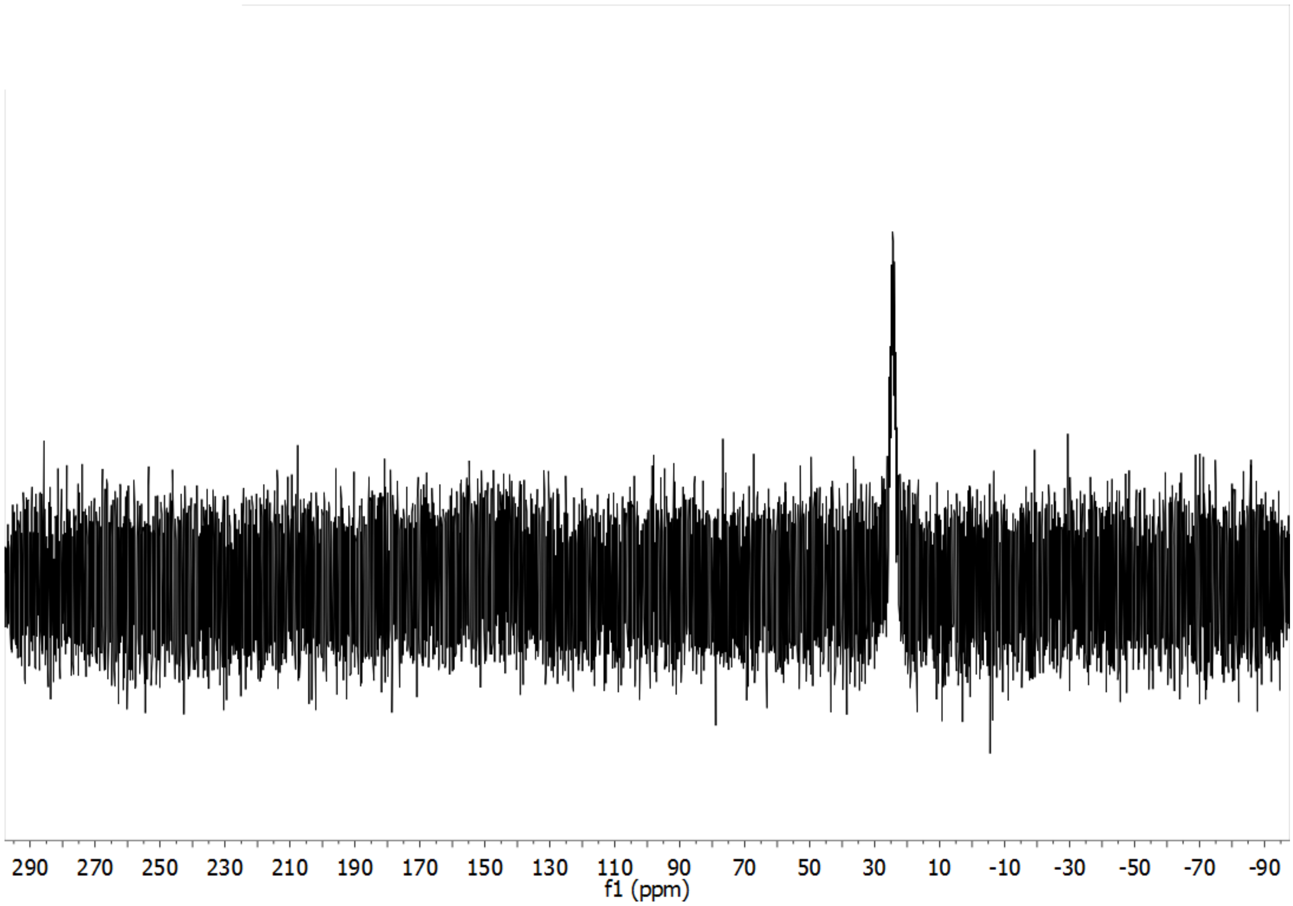

Supplementary Figure 16. ***$^{31}P\{^{1}H\}$ NMR spectrum of isolated $[\{Th(Tren^{TIPS})\}_2(\mu\text{-}PH)]$ (5).***

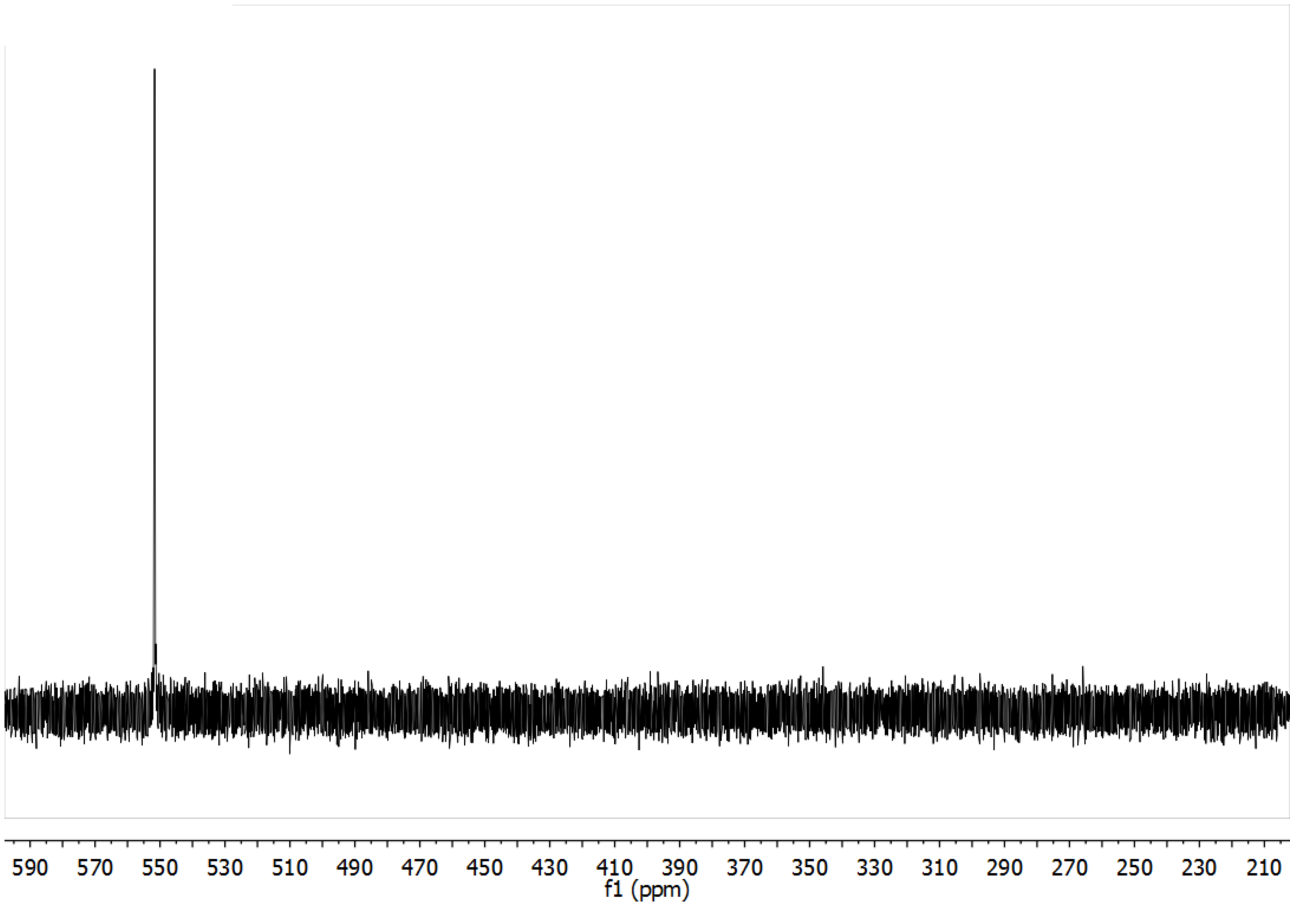

Supplementary Figure 17. ***$^{31}P\{^{1}H\}$ NMR spectrum of isolated $[\{Th(Tren^{TIPS})\}_2(\mu\text{-}P)][Na(12C4)_2]$ (6).***

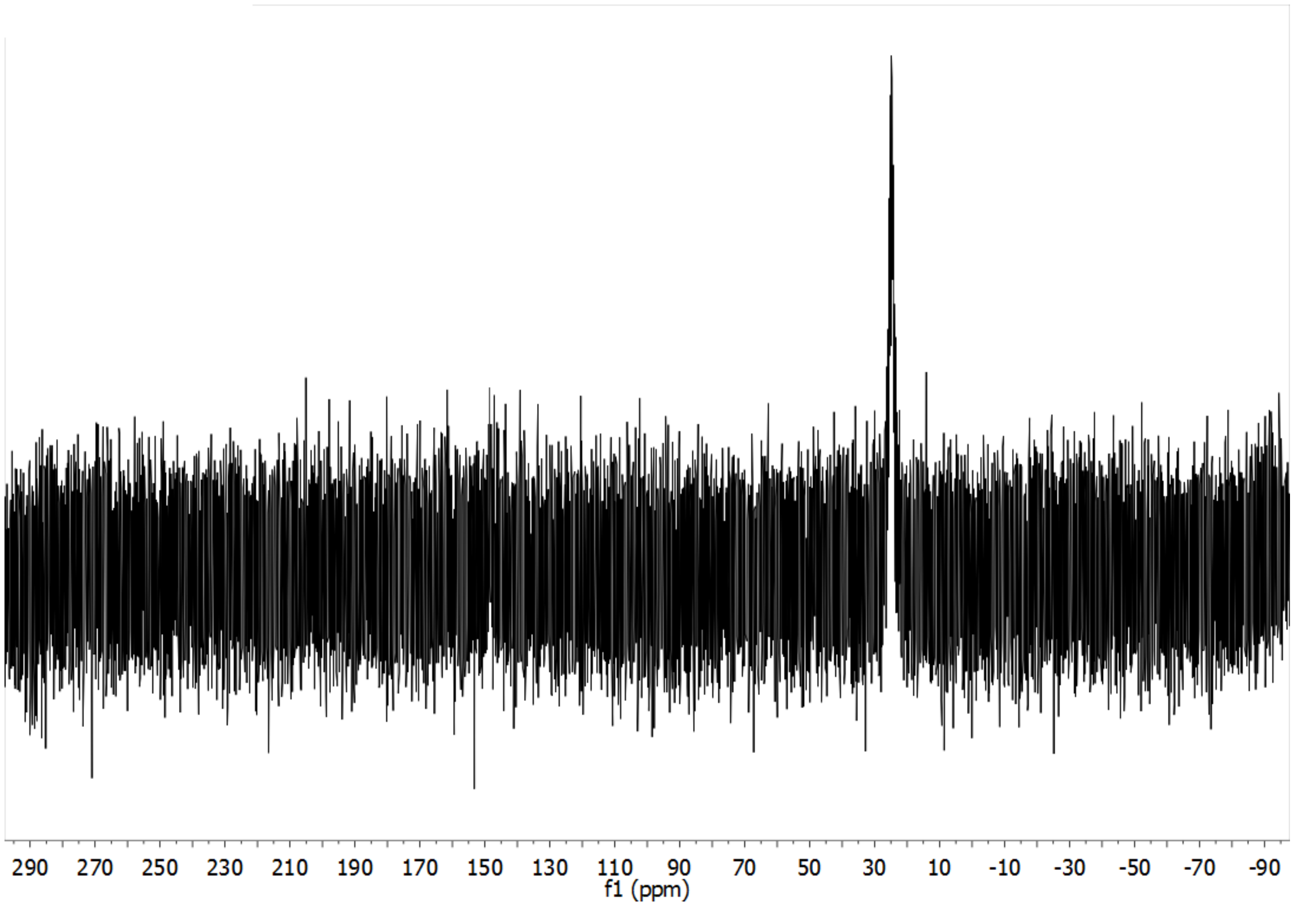

Supplementary Figure 18. ***^{31}P NMR spectrum of isolated [{Th(Tren^{TIPS})}$_2$(μ-PH)] (5).***

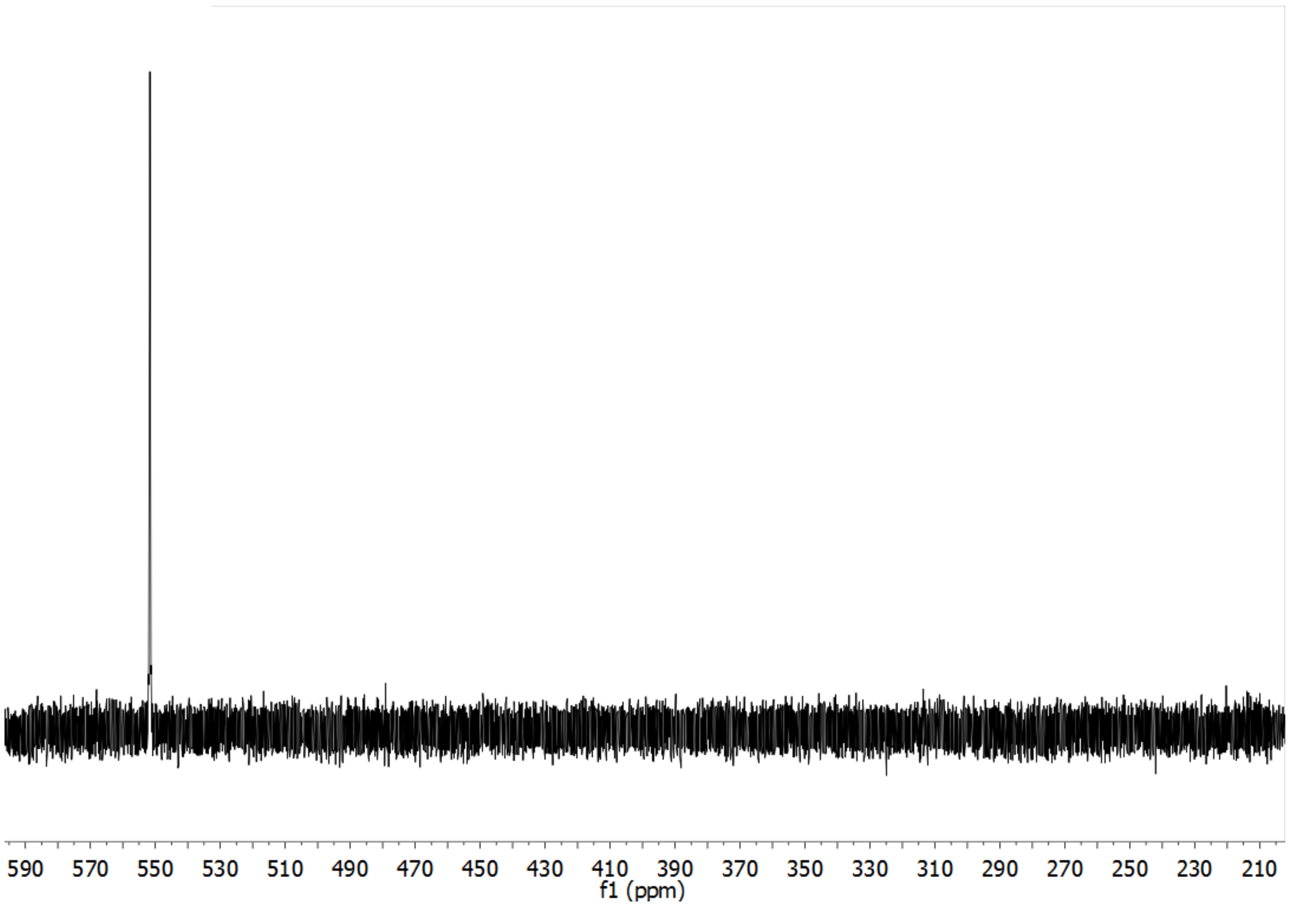

Supplementary Figure 19. ***^{31}P NMR spectrum of isolated [{Th(Tren^{TIPS})}$_2$(μ-P)][Na(12C4)$_2$] (6).***

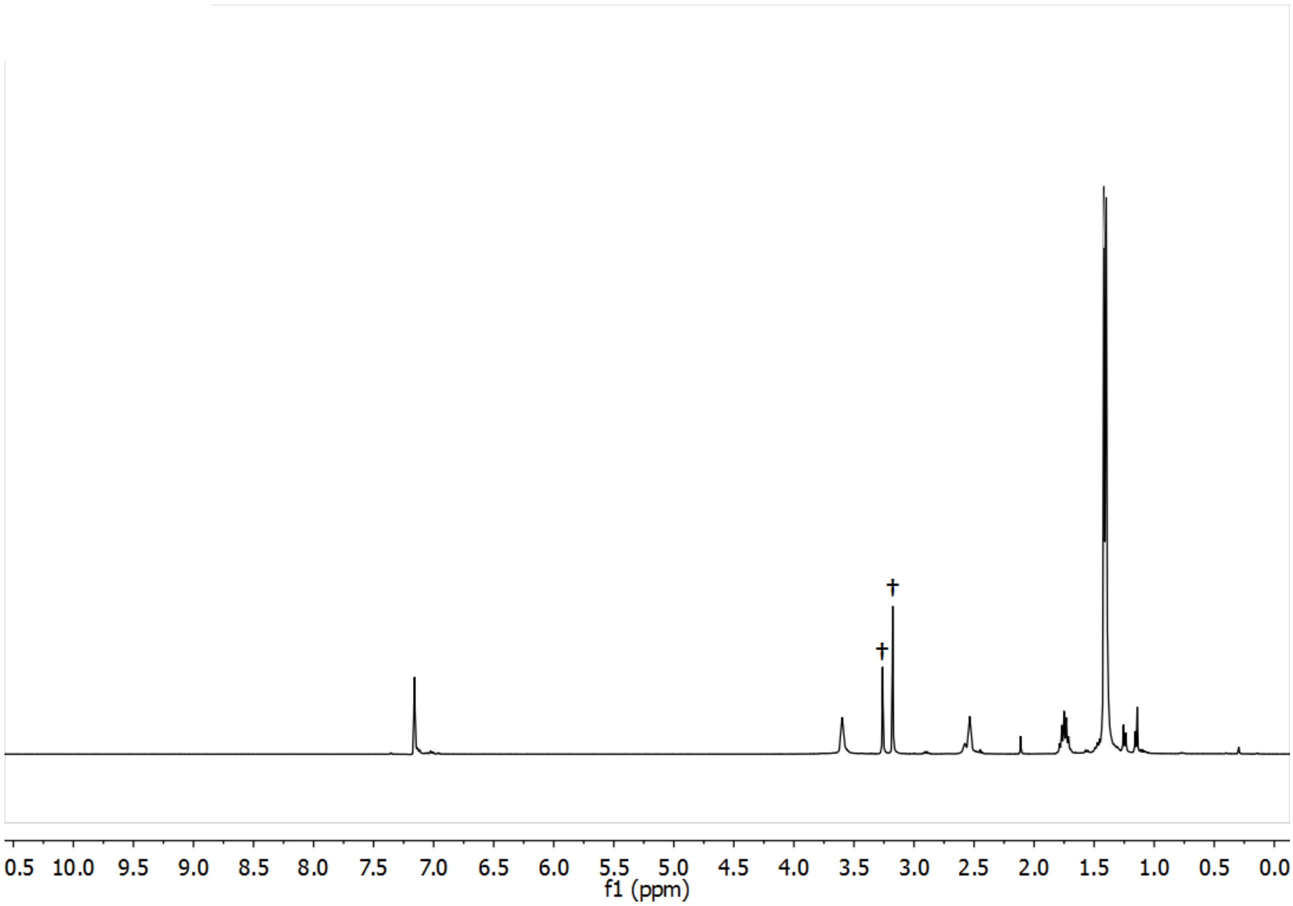

Supplementary Figure 20. ***^{1}H NMR spectrum of isolated [{Th(Tren^{TIPS})}$_2$(μ-PH)] (5). † = residual solvent resonance.***

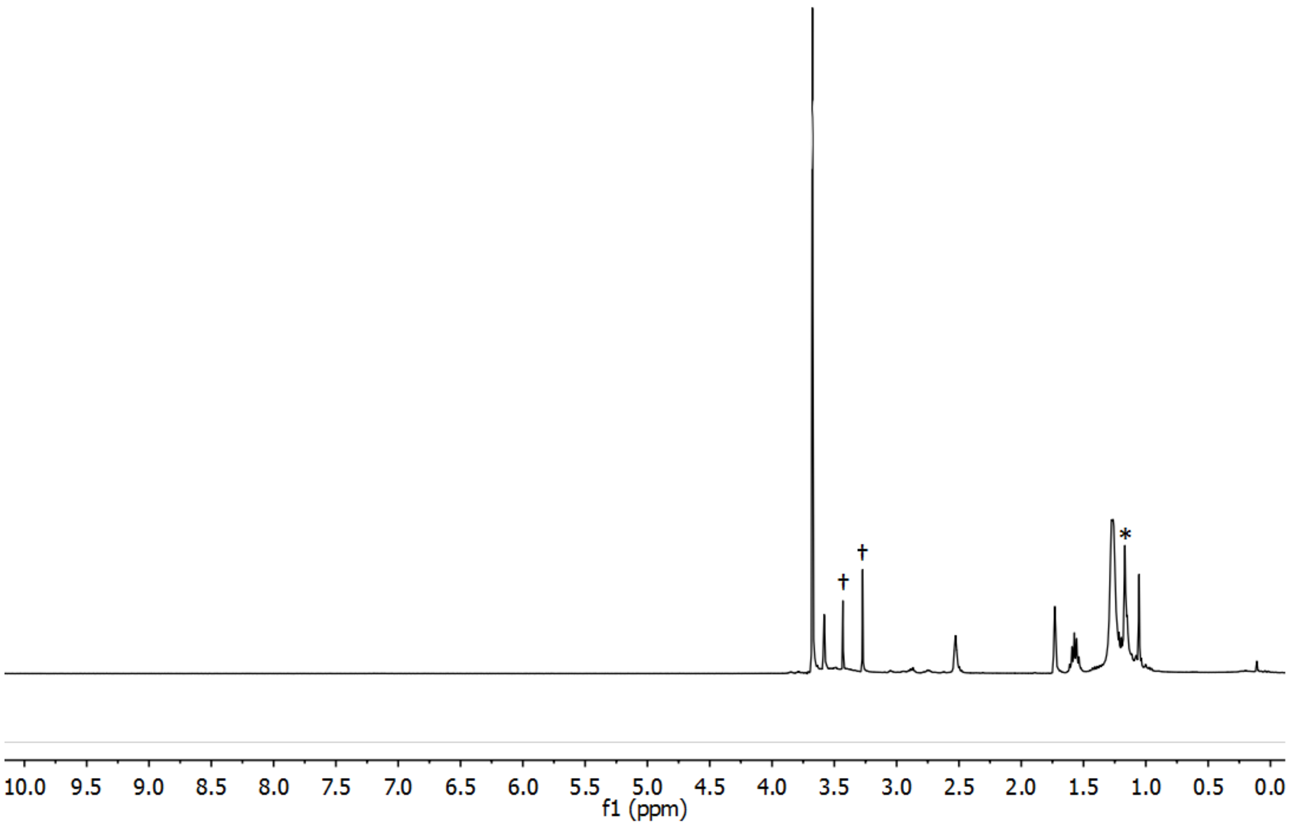

Supplementary Figure 21. ***^{1}H NMR spectrum of isolated [{Th(Tren^{TIPS})}$_2$(μ-P)][Na(12C4)$_2$] (6).***

**** = Tren^{TIPS}H$_3$ impurity, † = residual solvent resonance.***

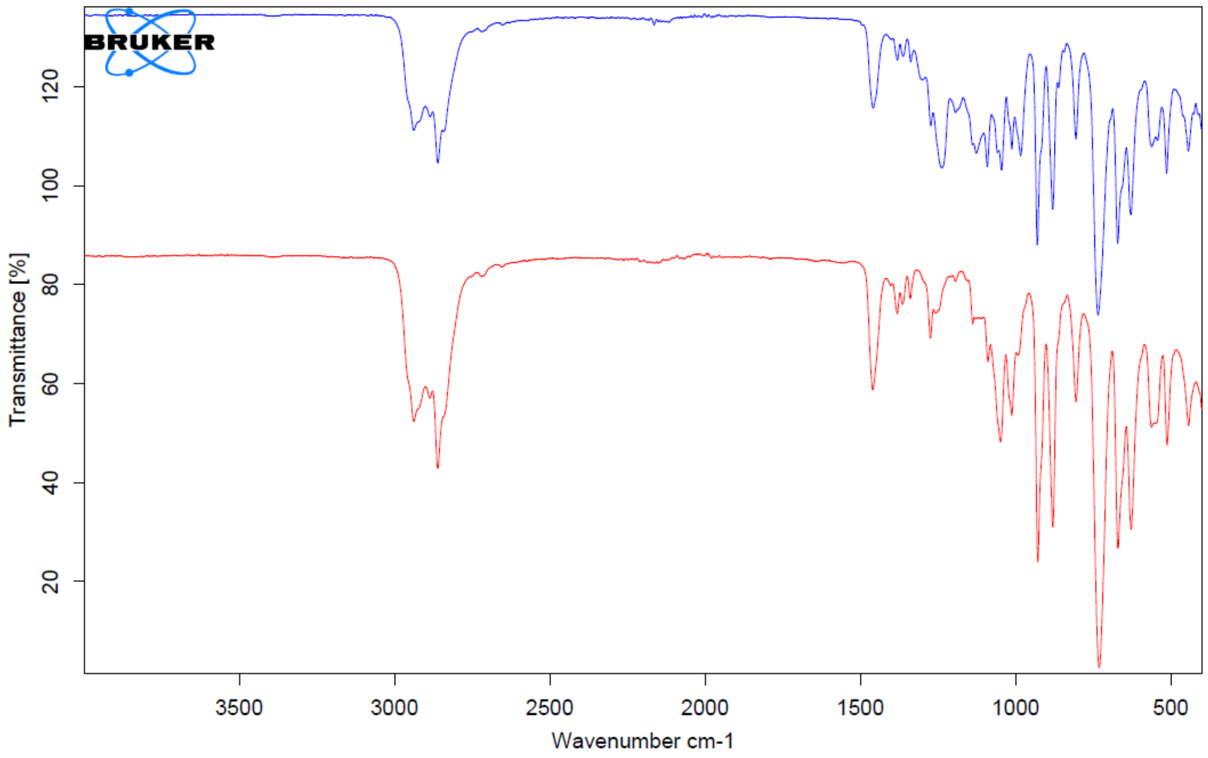

Supplementary Figure 22. ATR-IR spectrum of isolated [{Th(Tren^{TIPS})}$_2$(μ-PH)] (5, blue) and [{Th(Tren^{TIPS})}$_2$(μ-PD)] (5D, red). The transmittance scale is indicative and relative, not absolute.

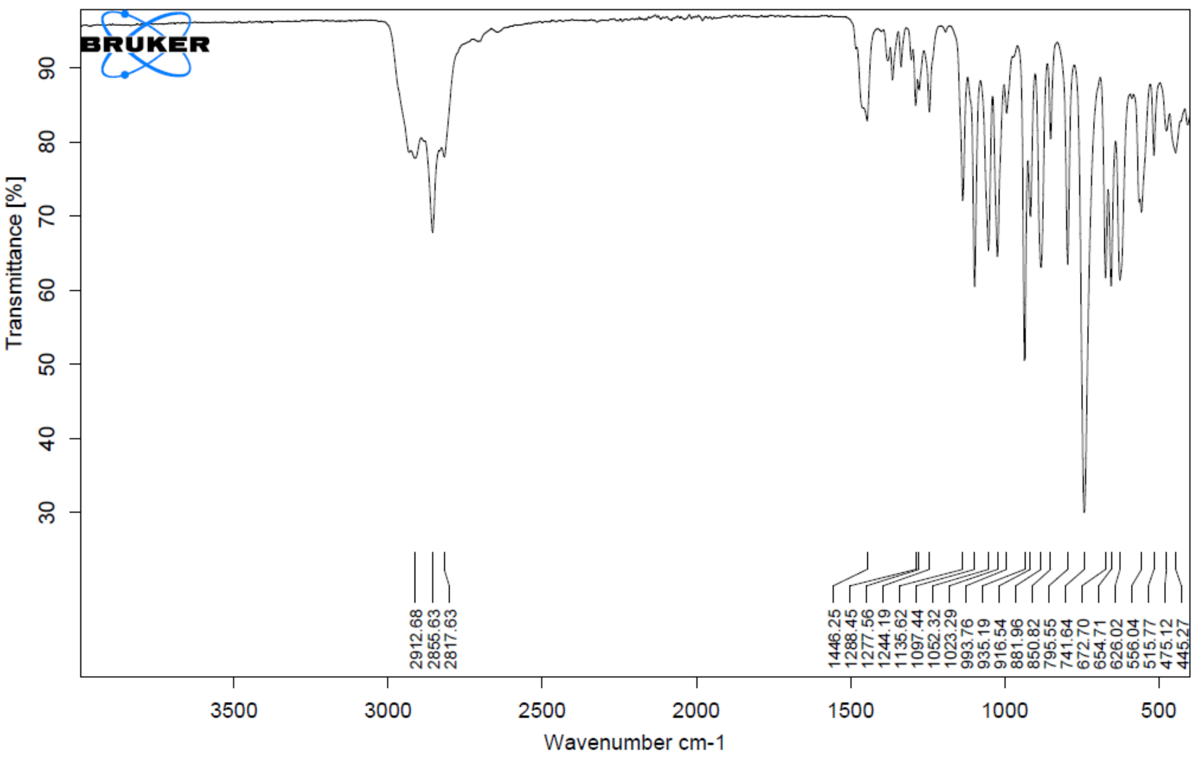

Supplementary Figure 23. ATR-IR spectrum of isolated [{Th(Tren^{TIPS})}$_2$(μ-P)][Na(12C4)$_2$] (6).

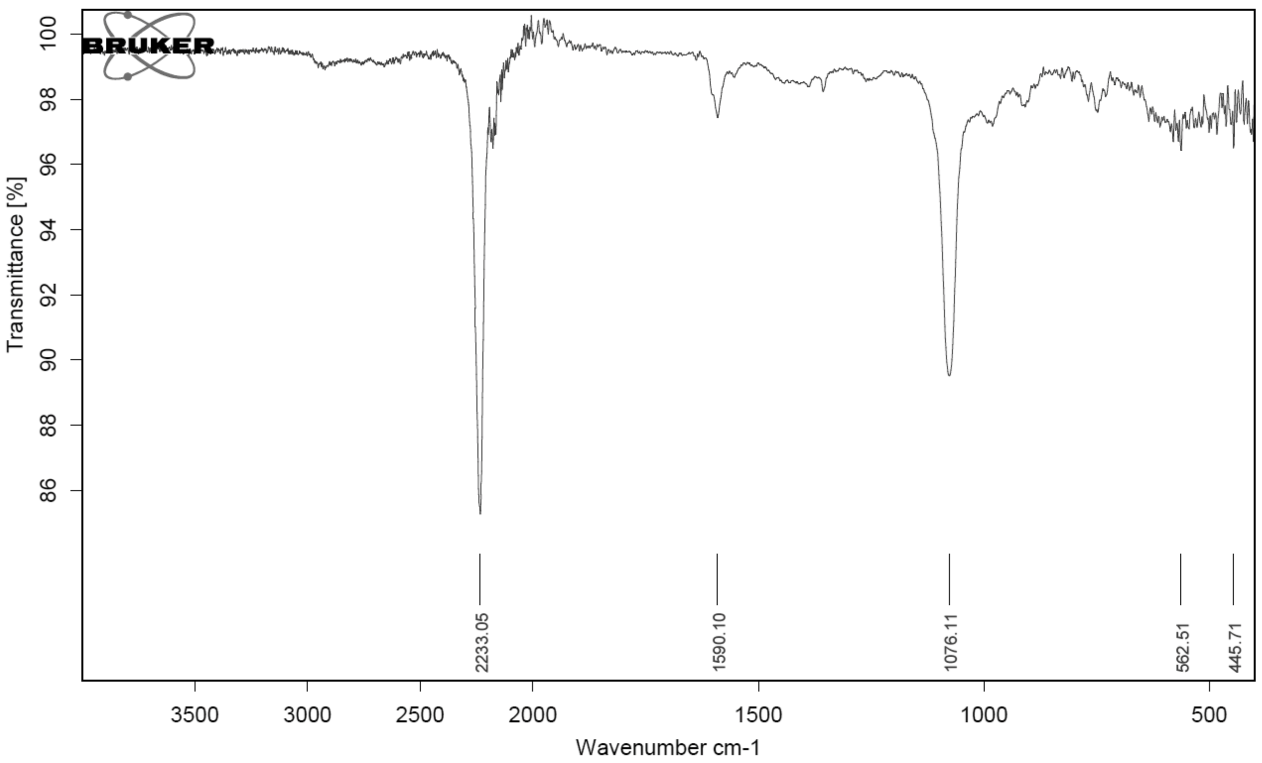

Supplementary Figure 24. ATR-IR spectrum of isolated $NaPH_2$.

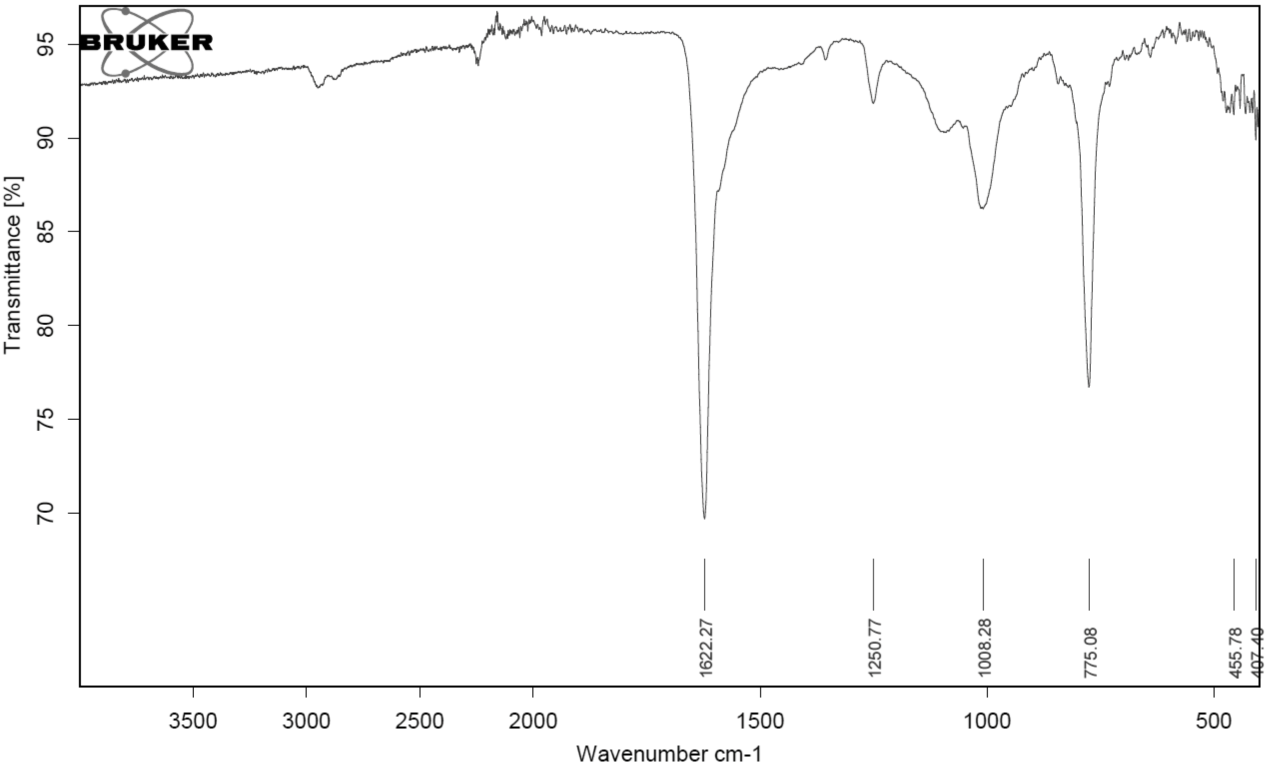

Supplementary Figure 25. ATR-IR spectrum of isolated $NaPD_2$.

Supplementary Tables

Supplementary Table 1. Final Coordinates and Single Point Energy of 2 after Geometry Optimisation

1.C	-1.449416	-0.960583	-6.271867
2.C	1.482870	1.003860	-5.043191
3.C	-1.842485	-0.979479	-4.778771
4.C	-2.978191	0.024529	-4.504166
5.C	0.898285	-2.243253	-4.073086
6.C	0.246362	-3.639594	-4.036250
7.C	0.646784	0.837110	-3.758385
8.C	-0.195731	2.101080	-3.503835
9.C	2.174218	-2.224047	-3.214495
10.C	-1.841553	-1.910993	-1.572127
11.C	-0.013642	5.053071	-0.842957
12.C	-2.918430	-1.307263	-0.675040
13.C	-2.483926	5.119438	-0.318019
14.C	3.907851	-3.203885	0.282032
15.C	-1.067386	4.991846	0.278030
16.C	0.561640	-4.563791	0.198443
17.C	-2.658070	1.689919	0.352253
18.C	3.070421	-2.019666	0.808428
19.C	-3.116063	0.415793	1.058249
20.C	4.014228	-0.948946	1.392992
21.C	1.132893	-4.342055	1.609821
22.C	-2.126254	-1.774025	1.617344
23.C	0.999779	3.401879	1.948379
24.C	1.508124	4.716990	2.575837
25.C	-0.896008	-1.438578	2.462548
26.C	0.226883	-5.021361	2.657548
27.C	-2.076542	3.714679	2.925353
28.C	1.411254	2.210406	2.831862
29.C	-2.154348	5.160145	3.461995
30.C	2.050424	-2.453575	3.857221
31.C	3.256804	-3.351915	4.206410
32.C	-1.859537	2.729178	4.090909
33.C	2.210066	-1.047542	4.464344
34.H	-2.324095	-1.176428	-6.908511
35.H	-0.676991	-1.706007	-6.511315
36.H	-1.063256	0.024144	-6.577483
37.H	0.842827	1.087818	-5.934793
38.H	-3.838693	-0.163060	-5.169712
39.H	2.172481	0.163756	-5.204922
40.H	2.092361	1.920715	-4.988447
41.H	1.197217	-2.042192	-5.119413
42.H	-0.632513	-3.711392	-4.693592
43.H	-2.248146	-1.989646	-4.576913
44.H	-0.851124	2.324596	-4.359150
45.H	-2.654167	1.061423	-4.678636
46.H	0.960428	-4.417018	-4.356452

47.H	-3.341528	-0.033159	-3.467552
48.H	2.876765	-3.016099	-3.523348
49.H	2.712148	-1.266780	-3.268305
50.H	-0.077358	-3.901678	-3.017090
51.H	0.453451	2.978570	-3.354848
52.H	1.389184	0.769758	-2.933851
53.H	-0.847792	2.017146	-2.618695
54.H	-2.348736	-2.299173	-2.471665
55.H	1.935425	-2.403850	-2.153865
56.H	-0.169018	5.939347	-1.481589
57.H	2.263527	2.518206	-1.426346
58.H	-0.072800	4.168909	-1.494261
59.H	-2.587204	6.059171	-0.886536
60.H	-3.396697	-0.481527	-1.219392
61.H	-1.408811	-2.797101	-1.075690
62.H	-2.696362	4.296305	-1.017362
63.H	4.684750	-2.841579	-0.410493
64.H	-2.857471	1.595838	-0.731434
65.H	1.012920	5.106305	-0.453546
66.H	-3.704489	-2.047578	-0.428938
67.H	1.155621	-4.076088	-0.586679
68.H	3.305759	-3.945641	-0.259314
69.H	0.505911	-5.637821	-0.046714
70.H	2.597284	-1.562532	-0.090373
71.H	-3.271009	5.115508	0.451211
72.H	-0.460975	-4.163735	0.122649
73.H	3.245021	1.859668	0.275026
74.H	4.704368	-0.579698	0.617971
75.H	-0.906422	5.869434	0.933033
76.H	-3.304298	2.516860	0.689052
77.H	4.420855	-3.726933	1.103984
78.H	-4.200008	0.232434	0.922660
79.H	1.529677	3.281875	0.982988
80.H	-1.951886	-2.737085	1.119942
81.H	2.110375	-4.860406	1.649141
82.H	3.475604	-0.078401	1.791049
83.H	1.288667	5.593098	1.949715
84.H	4.623609	-1.365483	2.208880
85.H	-2.925820	0.529146	2.133333
86.H	-3.036181	-1.874928	2.240549
87.H	0.070332	-6.084780	2.408202
88.H	-2.428608	5.882913	2.680566
89.H	-3.073124	3.490480	2.498225
90.H	2.600794	4.682203	2.720823
91.H	1.059611	1.230593	2.462655
92.H	-0.771045	-4.556112	2.701841
93.H	2.507640	2.140241	2.912822
94.H	-1.079515	-0.490673	3.002428
95.H	-0.804848	-2.205859	3.249974
96.H	1.055706	4.893470	3.564109
97.H	4.188315	-2.969490	3.760330
98.H	0.654164	-4.984384	3.669665

99.H	-1.196592	5.488666	3.894553
100.H	3.124374	-4.386334	3.855362
101.H	-1.744122	1.690069	3.748704
102.H	1.014038	2.305069	3.852153
103.H	-2.912258	5.237211	4.260520
104.H	3.087085	-0.523124	4.059003
105.H	1.166639	-2.907143	4.346066
106.H	1.332182	-0.417197	4.269813
107.H	-0.956077	2.985570	4.664894
108.H	-2.706954	2.756700	4.796750
109.H	3.414662	-3.392925	5.297655
110.H	2.343633	-1.106395	5.557850
111.N	-0.798040	-0.911836	-1.896918
112.N	-2.326603	-0.743050	0.565387
113.N	-1.218152	1.936902	0.613390
114.N	0.316958	-1.339333	1.616298
115.P	2.854525	1.288883	-0.978820
116.Si	-0.313077	-0.820236	-3.605186
117.Si	-0.851873	3.468012	1.440146
118.Si	1.608955	-2.500582	1.976672
119.Th	0.168775	0.210323	-0.104112

Energy: −636.89138585 eV

Supplementary Table 2. Final Coordinates and Single Point Energy of the Anion Component of 3 after Geometry Optimisation

1.C	0.112581	2.755946	-4.674541
2.C	1.374563	4.893884	-4.217583
3.C	0.903008	-3.674765	-4.008426
4.C	3.999208	2.258251	-3.476598
5.C	0.612201	3.715795	-3.576526
6.C	2.377492	0.323012	-3.372686
7.C	2.881471	1.587090	-2.651805
8.C	1.311495	-4.110941	-2.587730
9.C	2.735982	-3.621403	-2.261738
10.C	-1.270795	-1.360824	-2.326219
11.C	-1.375093	-6.084684	-2.050120
12.C	-1.602242	-4.609669	-1.660108
13.C	-2.529356	-0.736154	-1.708221
14.C	-2.084842	1.689622	-1.547624
15.C	2.413855	4.242605	-1.116613
16.C	-0.986875	2.572485	-0.940174
17.C	-2.694107	-4.517693	-0.575888
18.C	1.410682	5.228666	-0.486399
19.C	3.392261	3.716927	-0.050173
20.C	0.785914	-3.988677	0.458453
21.C	-3.030266	0.495094	0.383060
22.C	1.251682	-5.453020	0.592955
23.C	-2.454405	-0.303658	1.560153
24.C	-0.062843	-3.600599	1.681660

25.C	0.773830	2.962855	2.775050
26.C	-2.865669	2.873998	3.242609
27.C	0.742586	1.726789	3.691539
28.C	-2.299116	1.874784	4.270574
29.C	-1.788244	-1.844099	4.667995
30.C	-0.716208	-0.752238	4.863175
31.C	0.685886	-1.396143	4.851707
32.C	-3.462962	1.226183	5.046603
33.C	1.107063	2.150324	5.128645
34.H	0.949391	2.381734	-5.285029
35.H	-0.587754	3.264347	-5.362114
36.H	0.760865	5.378282	-4.999838
37.H	2.309523	4.564633	-4.698623
38.H	1.627909	-4.037020	-4.759364
39.H	3.633492	2.588273	-4.463213
40.H	-0.089127	-4.055821	-4.298565
41.H	-0.404062	1.877962	-4.260364
42.H	1.890003	0.553653	-4.331924
43.H	0.874794	-2.577569	-4.088501
44.H	4.827955	1.552543	-3.658448
45.H	3.217409	-0.360190	-3.582026
46.H	1.638579	5.668145	-3.482455
47.H	4.422843	3.139031	-2.971496
48.H	-0.289454	4.155802	-3.108570
49.H	3.437051	-3.877054	-3.076960
50.H	-1.602482	-2.136639	-3.039994
51.H	-0.692741	-6.190360	-2.905994
52.H	-0.774947	-0.593436	-2.954305
53.H	1.328303	-5.218056	-2.572962
54.H	1.657922	-0.250871	-2.768333
55.H	-2.006370	-4.109467	-2.562261
56.H	-2.329523	-6.569835	-2.326674
57.H	-1.816202	1.481518	-2.592264
58.H	-3.247793	-0.414467	-2.492465
59.H	2.775816	-2.531215	-2.115349
60.H	3.006516	4.802983	-1.865077
61.H	3.329559	1.235704	-1.700141
62.H	-3.068750	2.206620	-1.547176
63.H	3.126751	-4.073871	-1.339088
64.H	-0.949248	-6.665141	-1.215868
65.H	0.734332	5.676813	-1.231358
66.H	-1.074855	3.574616	-1.396193
67.H	-3.023203	-1.503309	-1.096414
68.H	-3.662494	-4.899059	-0.948301
69.H	4.214047	3.134727	-0.490337
70.H	1.881774	-5.776640	-0.248260
71.H	-2.850987	-3.486991	-0.226972
72.H	1.932638	6.056928	0.025603
73.H	-2.427967	-5.120659	0.306258
74.H	0.786371	4.724718	0.266357
75.H	-1.228667	2.728261	0.129806
76.H	-4.067333	0.171904	0.151631

77.H	3.841428	4.549627	0.521295
78.H	1.690044	-3.348030	0.490528
79.H	2.890614	3.056431	0.671542
80.H	0.394094	-6.143910	0.648427
81.H	-3.069986	1.550880	0.680972
82.H	1.837715	-5.590535	1.517679
83.H	2.665542	0.722328	1.008625
84.H	-2.450419	-1.377562	1.282153
85.H	-0.999991	-4.175074	1.734852
86.H	-0.337605	-2.533774	1.698943
87.H	0.558604	2.721558	1.723252
88.H	-3.186412	-0.229635	2.386203
89.H	0.494832	-3.787207	2.613433
90.H	-2.086435	3.333236	2.619926
91.H	-3.576436	2.378530	2.563343
92.H	1.766580	3.441143	2.796544
93.H	0.042769	3.723498	3.093074
94.H	1.529553	1.033319	3.333688
95.H	-3.419595	3.689101	3.743524
96.H	-1.677574	-2.326776	3.686597
97.H	0.975975	-1.729022	3.843769
98.H	-4.101085	0.612115	4.390198
99.H	-2.814073	-1.453442	4.731770
100.H	-1.731590	2.470944	5.013197
101.H	2.103131	2.624711	5.153823
102.H	-4.116243	1.999663	5.491582
103.H	1.469476	-0.701923	5.185407
104.H	-1.686869	-2.635181	5.432847
105.H	0.389973	2.888555	5.526228
106.H	0.717542	-2.276294	5.519532
107.H	1.128941	1.303895	5.829388
108.H	-3.113048	0.581027	5.865243
109.H	-0.874703	-0.301654	5.863726
110.N	-0.362421	-1.872818	-1.284704
111.N	0.349817	1.968375	-1.106256
112.N	-2.176577	0.398928	-0.825297
113.N	-1.105246	0.162956	1.942042
114.P	2.951160	-0.713340	0.920497
115.Si	1.520996	2.826351	-2.090647
116.Si	0.009409	-3.585555	-1.255408
117.Si	-0.883786	0.702093	3.591856
118.Th	0.439271	-0.104779	0.108479

Energy: −634.24761089 eV

Supplementary Table 3. Final Coordinates and Single Point Energy of 5 after Geometry Optimisation

1.C	0.014211	1.024519	-6.327927
2.C	-0.797856	-1.299559	-6.186392
3.C	1.622840	-0.829268	-6.172136

4.C	-2.308694	-4.790490	-5.818747
5.C	-3.518608	2.641007	-5.638202
6.C	-3.019359	5.086831	-5.301748
7.C	-2.417298	3.664144	-5.296533
8.C	0.487109	2.047234	-5.299699
9.C	4.684720	1.593054	-5.289162
10.C	-1.879331	-1.306613	-5.109286
11.C	2.201723	-1.639814	-5.016349
12.C	-0.050777	-5.123005	-4.733199
13.C	5.029725	0.167584	-4.815979
14.C	6.558178	0.050772	-4.622650
15.C	-1.537257	-4.806212	-4.481766
16.C	0.508530	5.404406	-4.373604
17.C	5.093595	-3.164143	-3.761821
18.C	-0.415672	4.859940	-3.264359
19.C	-3.841237	-3.254234	-3.160043
20.C	-4.543322	-4.598037	-2.874976
21.C	4.877834	-2.071775	-2.693133
22.C	-3.167004	1.407214	-2.338336
23.C	-4.327763	-2.179959	-2.174622
24.C	-2.429854	2.748916	-2.194512
25.C	3.133537	2.062158	-2.130444
26.C	0.365220	4.745471	-1.945600
27.C	-3.420686	3.851695	-1.770191
28.C	3.890916	0.754607	-1.838654
29.C	0.410947	-3.322057	-1.577246
30.C	-1.086883	-3.700363	-1.591038
31.C	4.204909	-2.684405	-1.455063
32.C	5.266831	1.077058	-1.222733
33.C	-1.285386	-5.160705	-1.133591
34.C	-5.274281	-0.221893	0.879259
35.C	1.911643	5.120164	1.416581
36.C	-3.852488	-0.168139	1.476249
37.C	-3.492258	1.299652	1.757966
38.C	3.106644	-4.054851	1.756273
39.C	-2.007340	4.786043	1.633885
40.C	-5.040681	-3.746116	1.931807
41.C	1.320452	3.790568	1.926692
42.C	4.680617	-0.383156	2.200802
43.C	2.452951	2.770755	2.136647
44.C	2.205882	-2.877243	2.177600
45.C	-3.634994	-3.183190	2.228559
46.C	0.742883	-3.345188	2.264173
47.C	-1.250472	5.241631	2.891901
48.C	-2.831693	-4.223032	3.035843
49.C	4.613848	-1.318457	3.418543
50.C	-2.261798	5.640956	3.987602
51.C	-5.094779	-1.188888	4.169147
52.C	5.304104	-0.647728	4.625476
53.C	-5.355173	0.241881	4.679881
54.C	1.166838	6.341479	4.895770
55.C	0.977273	4.810490	4.984279

56.C	-1.400097	2.383209	4.974277
57.C	-1.745505	-1.703240	4.938794
58.C	4.244464	-4.049132	5.301636
59.C	-5.185870	-2.187535	5.343046
60.C	2.986001	-3.151972	5.300764
61.C	2.304053	4.161070	5.422934
62.C	1.925074	0.020534	5.364561
63.C	1.747145	-4.019649	5.598266
64.C	-0.757613	1.644705	6.144721
65.C	-1.633960	-0.659034	6.045737
66.C	0.783112	-0.266684	6.334830
67.H	0.500553	1.167370	-7.312519
68.H	-1.209030	-1.057000	-7.185437
69.H	1.589777	-1.412386	-7.112967
70.H	-2.192810	-5.753726	-6.344811
71.H	-3.938956	2.834669	-6.640192
72.H	-1.067418	1.148748	-6.467706
73.H	-3.548310	5.278779	-6.251302
74.H	2.271159	0.040452	-6.341493
75.H	-0.357548	-2.303853	-6.240971
76.H	5.174763	1.818710	-6.252097
77.H	-1.934703	-4.010913	-6.500896
78.H	-1.683706	3.634803	-6.125596
79.H	-3.386712	-4.621258	-5.682941
80.H	0.382656	3.053914	-5.736035
81.H	7.088429	0.365058	-5.538342
82.H	4.765550	-0.519763	-5.643687
83.H	-3.155562	1.602714	-5.626267
84.H	0.057899	-6.038755	-5.340098
85.H	-2.708556	-1.950390	-5.448059
86.H	-2.255985	5.869255	-5.189023
87.H	0.452592	-4.312788	-5.284475
88.H	-0.000611	5.504692	-5.343901
89.H	3.172957	-2.051045	-5.337038
90.H	3.604359	1.748099	-5.422837
91.H	1.571448	1.915166	-5.138088
92.H	-2.314563	-0.293007	-5.038548
93.H	-4.353937	2.702149	-4.923749
94.H	1.559446	-2.521227	-4.840954
95.H	6.879959	-0.974428	-4.393568
96.H	5.553428	-2.779694	-4.684355
97.H	-3.752858	5.222873	-4.491923
98.H	5.035608	2.346526	-4.567277
99.H	1.382088	4.751435	-4.521122
100.H	0.896745	6.401465	-4.104073
101.H	-4.164347	-2.936114	-4.170170
102.H	4.142907	-3.644912	-4.038693
103.H	0.509687	-5.282631	-3.801651
104.H	-1.943920	-5.656855	-3.899483
105.H	6.915305	0.698229	-3.806581
106.H	-4.241345	-5.395937	-3.569061
107.H	5.752064	-3.961009	-3.375780

108.H	-5.638389	-4.488450	-2.957056
109.H	-1.216104	5.609218	-3.111543
110.H	-3.966444	1.457183	-3.090555
111.H	3.699654	2.723702	-2.801280
112.H	-2.514431	0.569254	-2.638564
113.H	-4.198259	4.008472	-2.533902
114.H	5.880386	-1.715803	-2.386666
115.H	0.924605	-3.512067	-2.529488
116.H	2.150615	1.918218	-2.612573
117.H	-3.896699	-1.196250	-2.394995
118.H	-5.426111	-2.081372	-2.203679
119.H	-4.335942	-4.950779	-1.852653
120.H	5.897905	1.646549	-1.922888
121.H	1.150427	3.979201	-2.012795
122.H	0.857635	5.699147	-1.691940
123.H	-2.923031	4.816548	-1.599582
124.H	-0.776082	-5.872439	-1.801263
125.H	3.183044	-3.017820	-1.687015
126.H	-3.623193	1.104371	-1.384270
127.H	-1.701052	2.623160	-1.369978
128.H	-4.052240	-2.434585	-1.139502
129.H	4.764053	-3.563004	-1.091762
130.H	2.943371	2.618242	-1.199584
131.H	5.819598	0.171010	-0.937898
132.H	-0.283860	4.477840	-1.100191
133.H	0.958349	-3.886277	-0.806113
134.H	0.565948	-2.265105	-1.288352
135.H	3.300371	0.215181	-1.070719
136.H	-3.936585	3.580486	-0.834587
137.H	-2.346031	-5.444717	-1.098121
138.H	-1.592204	-3.052364	-0.851993
139.H	4.133612	-1.970721	-0.622678
140.H	5.155391	1.690154	-0.313442
141.H	-5.359213	0.455477	0.013589
142.H	-0.871394	-5.310036	-0.122874
143.H	0.116351	1.483444	-0.104315
144.H	-5.545786	-1.225099	0.528163
145.H	2.491799	4.962911	0.492844
146.H	-3.145716	-0.522754	0.700307
147.H	-3.522644	1.889708	0.829173
148.H	2.786572	-4.469051	0.786509
149.H	-1.333122	4.587213	0.789526
150.H	1.134401	5.863624	1.191648
151.H	0.672440	3.391305	1.122269
152.H	2.981143	2.568060	1.192720
153.H	-5.637254	-3.093875	1.280123
154.H	-6.035033	0.093739	1.610467
155.H	-4.967471	-4.727120	1.431806
156.H	4.267635	-0.844544	1.293305
157.H	-3.112693	-3.067180	1.258678
158.H	-2.730217	5.551353	1.304182
159.H	0.396814	-3.751518	1.302541

160.H	2.255925	-2.112769	1.377572
161.H	4.159310	-3.757931	1.650633
162.H	5.721186	-0.090970	1.980558
163.H	2.594281	5.564895	2.157069
164.H	-2.484312	1.457778	2.177693
165.H	-2.571971	3.863214	1.826860
166.H	-4.187494	1.768167	2.468423
167.H	4.111914	0.539673	2.383103
168.H	3.063168	-4.875544	2.489098
169.H	-0.686481	6.157187	2.627384
170.H	-2.778694	-5.179723	2.489516
171.H	0.026866	-2.548234	2.529302
172.H	2.117803	1.787160	2.510337
173.H	-5.616336	-3.900558	2.858109
174.H	3.193567	3.130846	2.864028
175.H	5.196707	-2.227695	3.174856
176.H	0.611666	-4.132742	3.018475
177.H	-5.933963	-1.438487	3.488655
178.H	-1.803706	-3.894179	3.227383
179.H	-2.881826	6.489986	3.652400
180.H	-5.501908	0.958219	3.860333
181.H	-3.301680	-4.438743	4.008329
182.H	1.886597	6.616216	4.109037
183.H	6.366074	-0.446555	4.404275
184.H	-2.952970	4.814671	4.213410
185.H	4.217634	-4.786991	4.485066
186.H	3.116179	4.399791	4.718983
187.H	0.230494	6.876156	4.686123
188.H	4.840100	0.322309	4.860620
189.H	-2.363393	1.900161	4.728982
190.H	-1.781304	5.940710	4.930729
191.H	1.644967	-4.832295	4.862863
192.H	-5.006639	-3.227444	5.033813
193.H	-6.262811	0.274138	5.307605
194.H	-4.523048	0.616237	5.297281
195.H	5.176683	-3.477377	5.196983
196.H	-0.810464	-2.291406	4.915344
197.H	-1.663891	3.398215	5.313653
198.H	5.271872	-1.262396	5.537060
199.H	-2.532091	-2.424323	5.209058
200.H	2.010323	1.113053	5.227682
201.H	2.240244	3.065621	5.488215
202.H	-6.188485	-2.150389	5.803401
203.H	1.564578	6.735813	5.846857
204.H	0.809314	-3.445851	5.582377
205.H	0.246180	4.631338	5.797750
206.H	2.872247	-0.282831	5.840025
207.H	4.311630	-4.616511	6.246063
208.H	-4.464967	-1.948852	6.140489
209.H	3.100637	-2.454086	6.153462
210.H	2.617893	4.535007	6.412919
211.H	-2.552934	-0.058145	6.049790

212.H	1.828548	-4.495321	6.591016
213.H	0.199678	2.130514	6.375393
214.H	0.701574	-1.354197	6.460959
215.H	-1.385206	1.688495	7.056220
216.H	-1.528737	-1.122633	7.045843
217.H	0.964622	0.175323	7.333896
218.N	0.269346	-0.341068	-5.811491
219.N	-0.262537	1.887392	-4.026433
220.N	-1.317233	-1.734440	-3.801328
221.N	2.306236	-0.805160	-3.791391
222.N	-1.990648	-1.045308	3.628431
223.N	-0.493186	2.390684	3.794325
224.N	1.687085	-0.659906	4.065860
225.N	-0.487731	0.238165	5.760709
226.P	0.101742	0.040454	-0.006721
227.Si	-1.365455	3.240420	-3.713043
228.Si	3.980648	-0.476659	-3.308329
229.Si	-1.912113	-3.322156	-3.283115
230.Si	-3.594423	-1.380603	2.949195
231.Si	0.136735	4.005200	3.424629
232.Si	2.830795	-1.978655	3.753798
233.Th	0.212386	-0.160144	-2.991987
234.Th	-0.164397	0.229938	2.957438

Energy: −1257.97029814 eV

Supplementary Table 4. Final Coordinates and Single Point Energy of the Anion Component of 6 after Geometry Optimisation

1.C	4.718548	-0.008670	-6.472758
2.C	-4.062229	-1.081940	-5.976191
3.C	1.360255	1.311238	-5.302494
4.C	4.823404	0.124092	-4.938227
5.C	3.510576	-3.168925	-4.972028
6.C	2.552931	-1.960608	-4.928091
7.C	-3.015555	-1.256717	-4.859678
8.C	5.328358	1.535209	-4.578486
9.C	-2.174753	0.023161	-4.726052
10.C	1.163883	-2.414538	-4.451106
11.C	-6.427916	-2.486326	-3.885358
12.C	1.855440	0.894022	-3.904042
13.C	-5.098065	-3.093339	-3.388582
14.C	-1.923801	-4.234058	-3.261575
15.C	2.185417	2.144217	-3.071984
16.C	-5.398530	-4.024698	-2.196066
17.C	-1.751911	2.715614	-2.466997
18.C	-4.984265	0.326224	-2.325319
19.C	-2.123310	-2.929893	-2.463403
20.C	4.832644	-1.683675	-2.126229
21.C	-0.831172	5.033319	-2.112484

22.C	-4.553756	5.153319	-1.865683
23.C	5.996647	-0.928707	-1.483923
24.C	-6.104163	0.198816	-1.289928
25.C	-1.289658	3.736689	-1.413133
26.C	-2.168529	-3.195541	-0.944804
27.C	-3.733434	5.467100	-0.599339
28.C	1.612378	-3.566753	-0.751950
29.C	2.544357	4.770584	-0.446336
30.C	5.172954	1.936007	-0.226842
31.C	6.162271	0.853413	0.207417
32.C	-5.804019	2.055621	0.303758
33.C	-4.625245	6.176373	0.440867
34.C	4.559718	-5.352647	0.518597
35.C	3.528675	4.778615	0.733303
36.C	1.356766	-3.898618	0.730752
37.C	5.902491	-1.497877	0.912423
38.C	0.897157	-5.363120	0.882987
39.C	-6.131365	-0.238760	1.134070
40.C	-4.624942	2.601987	1.108054
41.C	-5.217123	-1.446971	1.341592
42.C	-0.988716	6.039078	1.466110
43.C	3.304502	6.023634	1.614977
44.C	-1.612015	4.633122	1.568709
45.C	4.045923	-4.927050	1.908395
46.C	4.776318	-1.510044	1.946660
47.C	6.276705	3.937254	2.576341
48.C	5.221696	-4.873244	2.906153
49.C	1.953005	2.611132	2.552198
50.C	-2.395831	4.507207	2.889492
51.C	-1.990171	-3.939524	2.818683
52.C	4.925809	3.363767	3.051745
53.C	-4.435200	-4.226596	3.354318
54.C	1.951915	1.110527	2.939654
55.C	-3.173373	-3.381024	3.624047
56.C	1.615558	3.506044	3.761883
57.C	2.088429	-3.207403	3.681004
58.C	-1.788353	0.922334	3.581454
59.C	-1.739519	-0.608675	3.734422
60.C	5.163714	2.193421	4.024591
61.C	1.744989	-4.516760	4.418839
62.C	-4.825468	-0.999987	4.649479
63.C	2.897001	-2.283027	4.611846
64.C	-5.006429	0.513086	4.879639
65.C	-1.093420	-0.988215	5.080663
66.C	-4.740029	-1.716889	6.014291
67.H	5.666349	0.290498	-6.956540
68.H	3.928398	0.639041	-6.885114
69.H	4.498698	-1.037619	-6.791618
70.H	-3.574430	-0.769864	-6.917239
71.H	-4.614092	-2.009806	-6.186992
72.H	2.442615	-1.594408	-5.967146
73.H	2.138356	1.859627	-5.858108

74.H	1.060344	0.448329	-5.914238
75.H	-4.800056	-0.301118	-5.729408
76.H	3.117131	-3.956400	-5.639409
77.H	-1.677899	0.275250	-5.678201
78.H	4.517063	-2.905546	-5.332500
79.H	6.326611	1.724433	-5.013080
80.H	0.485043	1.977988	-5.226980
81.H	-2.332871	-2.061846	-5.194138
82.H	4.653802	2.310577	-4.974641
83.H	-6.306596	-1.833301	-4.758925
84.H	0.815963	-3.291523	-5.025254
85.H	5.607945	-0.589016	-4.616362
86.H	-7.134677	-3.288188	-4.167280
87.H	0.407628	-1.625499	-4.565256
88.H	-4.714802	-3.734629	-4.208926
89.H	-2.804807	0.879105	-4.444033
90.H	-1.846785	-4.051885	-4.344046
91.H	3.622223	-3.621557	-3.974816
92.H	-1.397613	-0.065155	-3.954191
93.H	5.402011	1.693190	-3.492529
94.H	2.953566	2.768164	-3.551723
95.H	-6.920782	-1.897032	-3.096735
96.H	1.178085	-2.700975	-3.389650
97.H	-5.409456	0.121498	-3.324650
98.H	1.017810	0.388042	-3.383181
99.H	-2.746877	-4.948443	-3.105128
100.H	-1.084081	2.727246	-3.344348
101.H	5.189706	-2.100669	-3.084171
102.H	-0.993236	-4.737735	-2.951125
103.H	1.289254	2.765969	-2.927080
104.H	-2.773326	2.897668	-2.831601
105.H	-6.205008	-4.736869	-2.449822
106.H	-0.006412	4.820573	-2.813314
107.H	-3.929545	4.769107	-2.684466
108.H	-1.643598	5.490709	-2.699713
109.H	-5.067857	6.058391	-2.238350
110.H	-4.664583	1.385588	-2.361633
111.H	-1.229745	-2.303108	-2.640529
112.H	-4.525759	-4.614346	-1.886028
113.H	6.205841	-0.040449	-2.095270
114.H	2.560611	1.915653	-2.060657
115.H	-1.686424	1.680149	-2.088704
116.H	-5.332305	4.399814	-1.667488
117.H	-7.002571	0.784726	-1.574821
118.H	6.920837	-1.543307	-1.455123
119.H	4.598811	-2.564769	-1.499382
120.H	-5.737484	-3.455359	-1.315559
121.H	-0.468054	5.794013	-1.408116
122.H	-6.397951	-0.858034	-1.233022
123.H	0.899655	-4.103382	-1.399855
124.H	5.025763	1.851019	-1.320318
125.H	-2.968731	6.212367	-0.897264

126.H	2.766503	3.961141	-1.151208
127.H	2.622950	-3.821082	-1.099094
128.H	2.576553	5.724133	-1.002352
129.H	1.414893	-2.497298	-0.957116
130.H	-0.407692	3.288335	-0.917424
131.H	-5.832219	2.579267	-0.661264
132.H	-1.553419	-4.070489	-0.678018
133.H	-3.177547	-3.377602	-0.551022
134.H	7.162578	0.997679	-0.251241
135.H	-5.067478	7.093741	0.010269
136.H	3.741492	-5.554892	-0.185905
137.H	-1.701915	-2.356535	-0.389736
138.H	5.650031	2.922385	-0.079875
139.H	5.194935	-4.575765	0.063655
140.H	1.508549	4.626276	-0.104375
141.H	4.544775	4.873727	0.301545
142.H	5.170280	-6.271512	0.587334
143.H	0.006140	-5.550726	0.260267
144.H	5.939033	-2.484939	0.431877
145.H	-5.191690	-2.031423	0.402611
146.H	1.671429	-6.077205	0.561140
147.H	-6.771952	2.236042	0.816441
148.H	-0.300374	6.135813	0.615499
149.H	-5.466435	5.544984	0.768174
150.H	-7.178151	-0.544686	0.927256
151.H	3.422171	6.948810	1.022597
152.H	-4.067009	6.474004	1.339752
153.H	0.516319	-3.254047	1.048303
154.H	6.282290	0.917448	1.297392
155.H	6.890478	-1.315024	1.383277
156.H	-4.857412	3.640913	1.395673
157.H	-1.757136	6.822636	1.362457
158.H	6.155498	4.818967	1.929751
159.H	-0.779381	3.905628	1.627755
160.H	0.625609	-5.614750	1.916978
161.H	2.285036	6.041462	2.030369
162.H	-6.133715	0.350035	2.060925
163.H	6.863247	3.192982	2.014091
164.H	-2.145223	-3.806464	1.738714
165.H	-4.575527	2.047027	2.065033
166.H	1.146339	2.742266	1.806312
167.H	-5.701856	-2.110144	2.079638
168.H	3.377528	-5.738374	2.261326
169.H	-0.410182	6.275179	2.377027
170.H	-4.699582	-4.214339	2.285740
171.H	4.008346	6.082289	2.458842
172.H	5.998858	-4.162527	2.582951
173.H	4.718756	-0.500020	2.396093
174.H	5.710248	-5.861565	2.987465
175.H	1.402084	0.512679	2.181417
176.H	5.071990	-2.172775	2.776384
177.H	-3.232425	5.221512	2.942754

178.H	-2.305930	1.258142	2.667455
179.H	-2.807248	3.500266	3.027949
180.H	6.895804	4.244697	3.438762
181.H	-1.858985	-5.020346	3.003505
182.H	-1.043439	-3.444195	3.075006
183.H	2.956427	0.688474	3.080131
184.H	-1.076563	-0.983006	2.926695
185.H	1.547492	4.568378	3.487928
186.H	-4.265419	-5.282791	3.629688
187.H	5.600808	1.318995	3.516365
188.H	4.440654	4.165652	3.643887
189.H	4.905192	-4.579116	3.917775
190.H	-5.314143	-3.877538	3.917967
191.H	-1.737752	4.716745	3.750464
192.H	-0.776623	1.348733	3.534750
193.H	1.134909	-2.682315	3.479031
194.H	1.065333	-5.160998	3.844100
195.H	-5.029950	1.084789	3.940670
196.H	0.644071	3.217135	4.196182
197.H	1.406218	0.937648	3.880646
198.H	-5.752051	-1.363342	4.161977
199.H	3.100032	-1.311716	4.145204
200.H	2.649344	-5.105947	4.642793
201.H	4.236173	1.857914	4.508298
202.H	2.367284	3.418960	4.562326
203.H	-2.313188	1.397012	4.423325
204.H	5.866428	2.484382	4.826897
205.H	-2.927427	-3.494791	4.697784
206.H	3.863677	-2.727169	4.899836
207.H	1.250270	-4.303336	5.383110
208.H	-5.944480	0.723690	5.424360
209.H	-0.075903	-0.570289	5.160360
210.H	-1.010110	-2.076884	5.212835
211.H	-4.184085	0.922383	5.486824
212.H	2.340287	-2.092462	5.545911
213.H	-4.751199	-2.811821	5.917284
214.H	-1.672368	-0.593019	5.931406
215.H	-5.592671	-1.434329	6.658581
216.H	-3.821847	-1.444407	6.558825
217.N	3.649492	-0.807651	-2.277611
218.N	-3.849667	-0.561703	-1.983101
219.N	5.621357	-0.482164	-0.125811
220.N	-5.610700	0.613139	0.042434
221.N	-3.361177	2.479550	0.346849
222.N	3.890251	1.797145	0.495437
223.N	3.486920	-1.894062	1.327490
224.N	-3.857233	-1.025142	1.744955
225.P	-0.015436	-0.017672	-0.149362
226.Si	3.236050	-0.437326	-3.945037
227.Si	-3.590705	-1.880240	-3.109639
228.Si	-2.571899	4.015058	0.002115
229.Si	3.584098	3.076244	1.660817

230.Si	2.815178	-3.415603	1.891180
231.Si	-3.413418	-1.474792	3.384340
232.Th	2.745806	-0.204270	-0.152583
233.Th	-2.770696	0.195005	-0.001132

Energy: −1255.74153285 eV

Supplementary Methods

General Experimental Details

All manipulations were carried out using standard Schlenk techniques, or an MBraun UniLab glovebox, under an atmosphere of dry nitrogen. Solvents were dried by passage through activated alumina towers and degassed before use. All solvents were stored over potassium mirrors except for ethers which were stored over activated 4 Å sieves. Deuterated solvent was distilled from potassium, degassed by three freeze-pump-thaw cycles and stored under nitrogen. The compounds $[Th\{N(CH_2CH_2NSiPr^i_3)_2(CH_2CH_2NSiPr^i_2C[H]MeCH_2)\}]$ (**4**), $[HNEt_3][BPh_4]$, $NaCH_2Ph$, and $NaPH_2$ were prepared as described previously.[1-4] 12-crown-4 ether was dissolved in ether, stored over activated 4 Å sieves for 18 hours, then decanted and solvent removed *in vacuo* prior to use.

^1H, $^{13}C\{^1H\}$, ^{29}Si, and $^{31}P\{^1H\}$ NMR spectra were recorded on a Bruker 400 spectrometer operating at 400.1, 100.6, 79.5 and 162.0 MHz, respectively; chemical shifts are quoted in ppm and are relative to TMS (^1H, ^{13}C. ^{29}Si) and external 85% H_3PO_4 (^{31}P). FTIR spectra were recorded on a Bruker Alpha spectrometer with Platinum-ATR module. Elemental microanalyses were carried out by Mr Martin Jennings and Mrs Anne Davies at The University of Manchester School of Chemistry Microanalysis service; the data for $[Th(Tren^{TIPS})(OCH_2CH_2CH_2CH_2NEt_3)][BPh_4]$ are persistently low, which is a common problem with organosilicon-rich compounds.[5]

General Computational Details

Restricted geometry optimisations were performed for the full models of **2** and **5** and the full anion components of **3** and **6** using coordinates derived from the X-ray crystal structures. No constraints were imposed on the structures during the geometry optimisations. The calculations were performed using the Amsterdam Density Functional (ADF) suite version 2012.01.[6,7] The DFT geometry optimisations employed Slater type orbital (STO) triple-ζ-plus polarisation all-electron basis sets (from the ZORA/TZP database of the ADF suite). Scalar relativistic approaches were used within the ZORA Hamiltonian for the inclusion of relativistic effects and the local density approximation (LDA) with the correlation potential due to Vosko *et al*[8] was used in all of the calculations. Gradient corrections were performed using the functionals of Becke[9] and Perdew.[10] MOLEKEL[11] was used to prepare the three-dimensional plots of the electron density. Natural Bond Order (NBO) analyses were carried out with NBO 5.0.[12] The Atoms in Molecules analysis[13,14] was carried out with Xaim-1.0.[15]

Supplementary References

1. Gardner, B. M., Cleaves, P. A., Kefalidis, C. E., Fang, J., Maron, L., Lewis, W., Blake, A. J. & Liddle, S. T. The role of 5f-orbital participation in unexpected inversion of the σ-bond metathesis reactivity trend of triamidoamine thorium(IV) and uranium(IV) alkyls. *Chem. Sci.* **5**, 2489-2497 (2014).
2. Barker, B. J. & Sears, P. G. Conductance behaviour of some ammonium and partially substituted ammonium tetraphenylborates in 3-methyl-2-oxazolidone and 3-tert-butyl-2-oxazolidone at 25.deg. *J. Phys. Chem.* **78**, 2687-2688 (1974).
3. Bertz, S. H., Gibson, C. P. & Dabbagh, G. Preparation and reactivity of sodium organocuprates. *Organometallics* **7**, 227-232 (1988).
4. Klement R. in Brauer G. (ed.) *Handbuch der Präparativen Anorganischen Chemie*, 3rd edition, Ferdinand Enke Verlag, Stuttgart, 1975, Issue 1, page 516.
5. Hitchcock, P. B., Lappert, M. F., Maron, L. & Protchenko, A. V. Lanthanum does form stable molecular compounds in the +2 oxidation state. *Angew. Chem. Int. Ed.* **47**, 1488-1491 (2008).
6. Fonseca-Guerra, C., Snijders, J. G., Velde, G. Te & Baerends, E. J. Towards an order-N DFT method. *Theor. Chem. Acc.* **99**, 391-403 (1998).
7. Velde, G. te, Bickelhaupt, F. M., Van Gisbergen, S. J. A., Fonseca-Guerra, C., Baerends, E. J., Snijders, J. G., Ziegler, T. Chemistry with ADF. *J. Comput. Chem.* **22**, 931-967 (2001).
8. Vosko, S. H., Wilk, L. & Nusair, M. Accurate spin-dependent electron liquid correlation energies for local spin density calculations: A critical analysis. *Can. J. Phys.* **58**, 1200-1211 (1980).
9. Becke, A. D. Density-functional exchange-energy approximation with correct asymptotic behavior. *Phys. Rev. A* **38**, 3098-3100 (1988).
10. Perdew, J. P. Density-functional approximation for the correlation energy of the inhomogeneous electron gas. *Phys. Rev. B* **33**, 8822-8824 (1986).

11. Portmann, S. & Luthi, H. P. MOLEKEL: An interactive molecular graphics tool. *Chimia* **54**, 766-770 (2000).

12. NBO 5.0: Glendening, E. D., Badenhoop, J. K., Reed, A. E., Carpenter, J. E., Bohmann, J. A., Morales, C. M., Weinhold F. Theoretical Chemistry Institute, University of Wisconsin, Madison, WI, 2001; http://www.chem.wisc.edu/~nbo5.

13. Bader, R. F. W. *Atoms in Molecules: A Quantum Theory*, Oxford University Press, New York, 1990.

14. Bader, R. F. W. A bond path: A universal indicator of bonded interactions. *J. Phys. Chem. A* **102**, 7314-7323 (1998).

15. http://www.quimica.urv.es/XAIM.
